# Genome-Based Inference of a 50-Kilobase Collinear Synteny Block Containing Genes Predicted to Confer Antimicrobial Resistance and Conjugation Is Widespread Across a Putative Genomic Island in Alpha-Proteobacteria

**DOI:** 10.3390/microorganisms14102390

**Published:** 2026-10-19

**Authors:** Ciro Cubillas, Araceli Dávalos, Alejandro García-de los Santos

**Affiliations:** Programa de Ingeniería Genómica, Centro de Ciencias Genómicas, Universidad Nacional Autónoma de México, Avenida Universidad s/n Col. Chamilpa, Cuernavaca 62210, Mor., Mexico; cubillas@ccg.unam.mx (C.C.); davalos@ccg.unam.mx (A.D.)

**Keywords:** co-occurrence, Cu-P-type ATPases, gene clusters, copper transporters, antimicrobial resistance, collinear operon, genomic islands

## Abstract

Antibiotic-, biocide-, and metal-resistance genes co-occur in the genomes of multidrug-resistant bacteria. This relationship is more complex than mere physical co-localization. Instead, it arises from co-selection pressures that favor co-regulation and co-resistance. The genomic basis of this co-occurrence remains unclear. We predicted the co-occurrence of Cu(I)-ATPases with other metal-extrusion systems in *Methylobacterium extorquens*. In this study, we identified a more complex co-occurrence of Cu(I)-ATPases in the pathogen *Brucella anthropi* ATCC 49188. It comprises five collinear gene clusters spanning 48.5 kb and may be involved in multimetal homeostasis, redox homeostasis, conjugation, and the transport of miscellaneous molecules. We found highly homologous clusters in 43 strains across 3 alpha-proteobacterial orders, isolated from diverse habitats in different countries. Despite these geographical differences and phylogenetic distances, most orthologous genes remain syntenic. We called this gene arrangement a Collinear Synteny Block (CSB). In 20 species, these CSBs reside within an inferred ~110 kb genomic island, which may have contributed to their dispersion. CSBs co-occur with potential antibiotic-resistance genes scattered across the same replicon. This study will advance our understanding of the organization, evolution, and dispersal of resistance genes in bacteria. This genome-mining study is entirely predictive, and the proposed hypothesis requires experimental validation.

## 1. Introduction

Reducing the transmission and emergence of antimicrobial resistance genes is one of the major health challenges of the 21st century; effectively addressing this issue requires a comprehensive study of the mechanisms behind their evolution and spread. Numerous studies have shown that genes conferring resistance to antibiotics, biocides, and metals can co-occur, highlighting the need to investigate how bacteria organize their antimicrobial resistance genes into genomic clusters and how this organization may contribute to adaptation to multiple stresses.

Copper is an essential transition metal that serves as a cofactor in respiration, photosynthesis, and numerous redox reactions. However, at elevated concentrations, copper becomes a potent bactericidal agent. The antimicrobial properties of copper have been extensively investigated, with promising results involving free Cu ions, Cu-based surfaces, Cu nanoparticles, and metallo-antibiotics [1,2,3].

Bacteria have experienced major changes in metal availability since the Great Oxidation Event (GOE) approximately 2.3 billion years ago. This event, characterized by the emergence of oxygenic photosynthesis, dramatically reduced the availability of Fe, Mn, and Mo and increased the solubility and use of Cu and Zn, among others [4,5]. Today, bacteria have to contend with uncontrolled metal pollution in air, soil, clinical and aquatic environments and in livestock production systems [6]. In response to these selection pressures, cells have evolved a wide range of mechanisms to ensure a sufficient supply of metals for proper metalloenzyme function, thereby avoiding intracellular metal overload and its toxic effects [7].

Cu(I) extrusion is an essential and conserved mechanism for maintaining intracellular Cu(I) concentrations [8,9]. The Cu(I)-transporting P1B-1-subtype ATPases, also known as Cu(I)-ATPases or CopA, are integral proteins of the inner membrane. They use the energy from ATP hydrolysis to transport Cu(I) out from the cytoplasm [10,11]. The metal-responsive MerR-family transcription factor CueR regulates transcription of these ATPases under conditions of copper excess [12]. In Gram-negative bacteria, translocation of Cu from the cytoplasm to the periplasm also requires CopZ, a small chaperone that delivers cytoplasmic Cu(I) to a Cu(I)-P-type ATPase [13]. The final delivery of Cu(I) to the periplasm requires the periplasmic metallochaperone CusF, which binds and delivers Cu(I) to the periplasmic Cu-proteome. CusF is encoded within the operon *cusCFBA* [14]. The CusCBA proteins form a channel that pumps Cu(I) from the periplasm into the extracellular space, using the proton-motive force [15]. A schematic representation of the Cu homeostasis pathway is provided in [8].

Much of our current understanding of the function, genetic regulation, biochemistry, and structure of the proteins that maintain Cu(I) homeostasis has been derived from the characterization of individual Cu(I) resistance genes [8,16]. However, comparatively little is known about the co-occurrence of genes conferring resistance to multiple metals. This is important because gene positions in a genome and their functional associations among genes within operons, regulons, and gene clusters may contribute to co-regulation and co-resistance [17,18]. Thus, analyses of genomic context and synteny can provide valuable insights into the co-occurrence and organization of metal-resistance and other antimicrobial-resistance determinants.

We previously investigated the co-occurrence, organization, and genomic location of genes comprising the Cu-resistome in the order *Rhizobiales* (Alphaproteobacteria). Our analysis revealed an unusually high number of *copA* paralogues encoding Cu(I)-transporting ATPases across the genomes of several *Rhizobiales* genera, suggesting a potential role for these proteins in enhanced Cu(I) tolerance; however, the functions of many of these proteins remain unknown [19]. Consistent with this hypothesis, we later reported that the genome of the highly copper-tolerant bacterium *Methylorubrum extorquens* AM1 (*Rhizobiales*) contains five *copA* genes (*copA1-copA5*) scattered across chromosomal and plasmid loci, each in distinct genomic contexts [20]. Our group also reported clustering of the co-localized genes *copA3*, *copA4*, and *copA5*, along with *cusAB*, *cusF*, and *copZ*, into three separate Copper Homeostasis Gene Clusters (CHGC3-CHGC5). The co-occurrence of CHGC3, together with three potential operons coding proteins responsible for maintaining the balance of Ni/Co (*rcnAR*), Zn (*zntA*), and Pb/Zn/Cd (P-type ATPase), was termed the Multimetal Homeostasis Gene Cluster (MHGC) [20].

The co-occurrence or scattered distribution of homeostasis genes for different metals across species has not been comprehensively studied. Recent studies of two *Cupriavidus gilardii* strains isolated from an iron-oxide deposit in an underground tunnel at the Naica mine (Chihuahua, México) revealed that their metal resistance gene clusters are scattered across the two chromosomes [21]. A genomics approach can help elucidate the molecular mechanisms underlying the organization, evolution, and dissemination of antimicrobial-resistance genes. The large volume of genomic data and the availability of useful algorithms enable us to explore and propose robust predictions about the role that genome organization may play in metal homeostatic systems.

Here, we report the identification of a multispecies heavy-metal translocating P-type ATPase (Ms-HMT-P-type ATPase). This protein is an identical P-type ATPase found in 43 bacterial genomes from strains isolated from diverse habitats worldwide. All 43 sequences are annotated with the same RefSeq (WP_012092676.1), have identical amino acid sequences, and are identical in length. We classified 36 strains within the order Rhizobiales, 5 strains within the order Geminicoccales, and 2 strains within the order Rhodobacterales. Because identical P-type ATPases have been found in distantly related bacterial species, suggesting a gene-transfer event, we investigated whether these 43 identical Ms-HMT-P-type ATPases share conserved genomic organization and potential functions and examined their distribution and evolutionary history.

Sequence analysis revealed that the Ms-HMT-P-type ATPase is a Cu(I)-transporting P1B-1-subtype ATPase predicted to confer copper resistance. This Cu(I)-ATPase is part of a Multispecies–Multimetal Homeostasis Gene Cluster (Ms-MHGC), in which at least 10 syntenic genes co-occur and are predicted to detoxify Cu(I), Co/Zn, and Cd. This cluster also contains a potential multidrug resistance transporter (MFS). The neighborhood analysis indicates that these Ms-MHGCs are collinear with four gene clusters: The Redox Homeostasis Gene Cluster (RHGC), the Conjugation Gene Cluster (CGC), the Miscellaneous Transporter Gene Cluster (MTGC), and the Arsenic Resistance Gene Cluster (ARGC). We propose a functional interrelation among five gene clusters. Together, these five gene clusters form a collinear synteny block (CSB) [22] spanning ~50 kb. In many clinical strains of *Brucella anthropi* (Rhizobiales), this CSB is located within a putative Genomic Island (GIBan) spanning 109.24 kb on chromosome 2. Numerous potential multidrug resistance transporters (MFS and RND superfamilies) were found scattered on chromosome 2.

The persistence and distribution of this putative resistance-associated CSB may reflect selective pressures imposed by environmental pollution, which can increase the co-occurrence of antibiotics, metals, and diverse biocides. Selection pressure from these agents may have increased multiresistance. This novel CSB provides new insights into the organization, evolution, and potential dissemination of resistance-associated genes in bacteria.

## 2. Material and Methods

### 2.1. Microbial Genome-Mining Tools Were Used to Identify the Ms-HMT-P-Type ATPase

We aimed to understand the distribution of Cu homeostasis genes within the order Rhizobiales. Using the putative Cu(I)-ATPase WP_116655330.1 from the highly copper-tolerant *M. radiotolerans* MLP1 [23] as a QuickBLASTP v2.15 query with default parameters [24], we identified the Ms-HMT-P-type-ATPase (WP_012092676.1, Alphaproteobacteria). We limited the search to Rhizobiales (Taxid: 356), excluding genomes from the *Methylobacteriaceae* family and sequences from uncultured/environmental samples.

We defined the diversity of Alphaproteobacteria species harboring identical Ms-HMT-P-type-ATPase using the Identical Protein Groups (IPG), a bioinformatic resource available at NCBI (https://www.ncbi.nlm.nih.gov/ipg/, accessed on 10 January 2026) [24]. It groups protein sequences that are 100% identical by amino acid residue and length under a single non-redundant protein accession number. The IPG contains proteins shared among multiple strains within a single species or clusters of identical proteins found in the genomes of different species, genera, families, or even superkingdoms [24]. We accessed the IPG from the “Identical Proteins” link in the GenePept record’s top menu. The IPG displays a downloadable table of organisms sharing identical proteins, a tree showing their relative taxonomic positions, and links to obtain the CDS region in nucleotide and genome assembly formats. Other useful NCBI bioinformatics tools used in this analysis were Graphics and CD-Search. We used the Graphics tool to quickly visualize the genes encoding the Ms-HMT-P-type ATPase WP_012092676.1 and its gene context. This resource was accessed by clicking on the “CDS region in nucleotides” link in the second column of the IPG table. The CD search interface to the Conserved Domain Database v3.21 (NCBI) identifies protein domains and signature motifs involved in binding or catalysis and is also useful for predicting the putative functions of protein sequences [25]. We inferred putative protein functions using BLASTP v2.17.0 comparisons, CD-Search, and co-occurrence of functionally related proteins via neighborhood gene analysis. We used a default threshold of 30% identity, 50% query coverage, and an E-value of 0.01 for BLAST and CD-Search.

### 2.2. Amino Acids/Nucleotide Multiple Sequence Alignments (MSAs) and Protein Topology of Ms-HMT-P-Type ATPase (WP_012092676.1) Identical Proteins

We used the Ms-HMT-P-type ATPase (WP_012092676.1) as a query in CD-Search (NCBI). The search result indicated that the Ms-HMT-P-type ATPase is similar to the human copper-transporting ATPases ATP7A and ATP7B in the CD02094 group. Because the default alignment displayed by CD-Search does not include bacterial homologs, we aligned the Ms-HMT-P-type ATPase amino acid sequence with Cu(I)-ATPases from Legionella pneumophila, Helicobacter pylori, Nitrobacter vulgaris, Drosophila melanogaster, and Candida albicans, all of which are included in the CD02094 group. Figure S1 shows that all these sequences conserve the three invariant amino acid signature motifs involved in Cu(I) binding found in TM helices 6, 7, and 8, as previously reported. We generated the MSA using MUSCLE v5.3 (accessed on 15 March 2026) [26] with maxiters set to −32. The translation of DNA into a protein sequence was performed using the Translate tool in Expasy [27]. The presence of 8 transmembrane helices was predicted by TOPCONS 2.0 web server (accessed on 10 April 2026) [28]. The metal transport motif CXC and the Cu(I) binding motifs, YN(X)_4_P and MSXST [29] were manually searched in the predicted transmembrane TM-6, TM-7, and TM-8, respectively.

The nucleotide FASTA sequences encoding the 43 multispecies P-type ATPases were collected from the nucleotide accessions IPG output Table (Table S1) and then aligned with Clustal Omega v1.2.4 (accessed on 20 March 2026) [30] at The Job Dispatcher, EMBL-EBI, using default settings.

### 2.3. Genomic Context Analysis and Definition of Gene Clusters

We initially visualized the context analysis of Ms-HMT-P-type ATPase WP_012092676.1 using the “Graphics” resource in NCBI via manual searches (Table S3). This observation revealed a putative organization in distinct functional clusters. To confirm this organization, we applied the following criteria: first, the DNA within a putative gene cluster must encode functionally related proteins. Second, these genes may have a putative operonic arrangement; third, they must be bordered by functionally unrelated intermediate genes, often encoded on a distinct DNA strand, most of which are functionally unrelated or lack conserved domains (hypothetical or DUF sequences; see Table 1). We used the Comparative Gene Clusters Analysis Toolbox (CAGECAT) v1.0 (accessed on 20 May 2026) to search for homolog clusters [31]. We used the proteins encoded in the five gene clusters of *Brucella anthropi* ATCC 49188 as queries for the CAGECAT program. We progressively used each gene cluster as a query for cblaster, adding one extra cluster to the prior set (i.e., MHGC + RHGC, then MHGC_RHGC + CGC, and so on) until we reached all 5 clusters and ~50 kb (CSB). This approach provided genomes carrying the closest homologous clusters across genomes, as shown in Figure 1, Figure 2, Figure 3, Figure 4 and Figure 5. We unlocked the “find intermediate genes” option and used the advanced settings for cblaster, including at least 30% homology, a maximum e-value of 0.01, and a minimum coverage of 50% for filtering; for the clustering settings, we set the maximum distance between genes to 20 kb and the minimum percentage of query genes present in clusters to at least 40% (default advanced settings). We used the cblaster search output for the clinker analysis, allowing us to compare gene cluster identity values with the clinker synteny results, as shown in Figure 1, Figure 2, Figure 3, Figure 4 and Figure 5.

### 2.4. Identification and Visualization of GIs Spanning the ~50 Kb CSB

Since the conserved region of highly identical proteins found in *Brucella anthropi* ATCC 49188 spanned ~50 kb, we used IslandViewer4 [32] to determine whether this CSB was located within a putative genomic island. IslandViewer4 combines three approaches, including: SIGI-HMM, which analyzes codon usage; IslandPath-DIMOB, which is based on dinucleotide sequence composition bias and the presence of mobility genes; and IslandPick, a comparative genomics approach [33]. IslandViewer4 has already precomputed genomes and generates a circular plot that displays putative GIs and the methods used to predict them. We searched for the WP_012092676.1 (OANT_RS16725) gene to establish whether it belonged to a GI. This search also provided a linear map showing the location of the OANT_RS16725 gene and a list of adjacent proteins within the GI. We downloaded GIs containing the entire ~50 kb synteny block, or partial versions missing one or more gene clusters, into Excel for further analysis.

### 2.5. Searching for GI Integration Sites and the Resulting Direct Repeats

Numerous GIs are inserted immediately downstream of a tRNA gene on one end, and a direct repeat (16–20 bp of the tRNA) is usually found on the other end. An integrase gene is also found at one end of the GIs [34,35]. To identify the potential integration site, we began with a visual inspection of the GI ends predicted for *B. anthropi* ATCC 49188 and used NCBI-Graphics to search for an integrase and a tRNA-encoding gene as a potential integration site. We also searched for putative direct repeats (DRs) flanking the GI created during island integration, using the Sequence Repeat Finder in DNA/RNA Repeat Finder|Cellerna Bioscience. We included 500 nucleotides upstream and downstream of the predicted GI start and end, assuming the DRs would be located near the tRNA at the integration site. We further manually mapped the identified DRs to pinpoint the position closest to the tRNA gene using Graphics-NCBI.

### 2.6. Searching for Multidrug Resistance Genes

To determine whether potential multidrug efflux pumps belonging to the Major Facilitator Superfamily (MFS) and the Resistance-Nodulation-Cell Division (RND) superfamily co-occurred with the genomic island harboring the five gene clusters, we searched *B. anthropi* ATCC 49188 Chromosome 2 using IslandViewer4. The toolbox panel includes an option to search for a gene. When we entered the terms MFS or RND, the software retrieved transporters annotated as MFS or RND and displayed a localization map alongside the genomic islands. We estimated the similarity of these proteins to experimentally tested efflux pumps using CARD/RGI BLASTP, a resource that predicts similarity between a query sequence and the Comprehensive Antibiotic Resistance Database (CARD) [36].

Table S5 lists the databases, software versions, access dates, parameters used, and websites.

## 3. Results

### 3.1. Identification of a Set of Identical Ms-HMT-P-Type ATPase Sequences Led to the Discovery of the Ms-MHGC

An Ms-HMT-P-type ATPase (WP_012092676.1) was identified during a Quick Protein BLAST search while studying the distribution of Cu(I) ATPases within the order Rhizobiales, excluding the family Methylobacteriaceae, as specified in the Materials and Methods section. According to the RefSeq non-redundant proteins documentation of NCBI (RefSeq non-redundant proteins), the prefix “MULTISPECIES” and the organism name in square brackets “[Alphaproteobacteria]” indicate that two or more 100% identical heavy metal translocating P-type-ATPases in terms of amino acid residues, length, and RefSeq have been annotated in the genomes of two or more Alphaproteobacteria strains. The number of identical proteins and the species harboring these P-type ATPases are shown in detail at the top of the GenePept record, which was provided by the Identical Protein Groups (IPG) resource (www.ncbi.nlm.nih.gov/ipg/). Based on the IPG record (accessed in 10 January 2026) and after removing redundancy between protein IDs (WP or INSDC), we identified 43 strains in three orders of the Alphaproteobacteria class (Rhizobiales, Rhodobacterales, and Geminicoccales orders) that all share the MULTISPECIES heavy metal translocating P-type ATPase [Alphaproteobacteria] with NCBI RefSeq (WP_012092676.1), with identical amino acid sequences and lengths (795 amino acids) (Table S1). We validated the taxonomic assignments using the NCBI taxonomy tool and through comparison with the most recent phylogeny of Alphaproteobacteria.

### 3.2. The Ms-HMT-P-Type ATPase Shares the Invariant Amino Acid Motifs of Cu(I) Translocating P-Type ATPases

Since P-type ATPases can transport various divalent cations, we used NCBI’s conserved domain search (CD-Search) to infer the putative cation translocated by the Ms-HMT-P-type ATPase. This search showed that the Ms-HMT-P-type ATPase is similar to Cu(I) ATPases, such as the human copper-transporting ATPases ATP7A and ATP7B, which are grouped in the CD02094 sequence cluster. Cu(I) ATPases share three key amino acid signature motifs (CXC, YN(X)_4_P, and MSXST) that are necessary for binding and transporting Cu(I) [29]. The potential Cu(I) specificity was reinforced by topology analysis using TOPCONS, which predicted the presence of eight transmembrane helices (TMHs), which is characteristic of P_1B_-type ATPases [28]. Further evidence was obtained by aligning the amino acid sequence of the Ms-HMT-P-type ATPase with homologous CopA from distantly related bacteria and eukaryotes such as *Legionella pneumophila*, *Helicobacter pylori*, *Nitrobacter vulgaris*, *Drosophila melanogaster*, and *Candida albicans*, all of which are included in the CD02094 group. This alignment revealed that the Ms-HMT-P-type ATPase conserves the three invariant amino acid signature motifs involved in Cu(I) binding found in TM helices 6, 7, and 8, as previously reported [29] (Figure S1). Based on these predictions, the name Ms-HMT-P-type ATPase was changed to Ms-Cu(I) ATPase. Because no identical Ms-Cu(I)-ATPases have been reported in distantly related species from unrelated environments, we explored their presence, genomic context, distribution, and evolutionary implications.

### 3.3. Identical Ms-Cu(I)-ATPases Are Encoded by the Genomes of Distantly Related Species from Diverse Habitats Around the World

According to the NCBI taxonomy database and based on the most recent genome-based taxonomic study of type strains of Rhizobiales, we inferred that the 43 strains sharing the same Ms-Cu(I)-ATPase1 WP_012092676.1 can be classified into three orders within the class Alphaproteobacteria [37]: 36 in Rhizobiales, two in Rhodobacterales, and five in Geminicoccales (Table S1). We inferred from the phylogenetic tree that the 36 strains are spread across seven distantly related families [38]. Notably, 21 of these strains belong to the *Brucellaceae* family. Among these, 12 strains are different strains of *B. anthropi.* Table S1 also highlights significant differences in the habitats and countries where these 43 strains were collected and isolated. Using information from BioSamples (NCBI) and published data, 43 bacteria with identical Ms-Cu(I)-ATPases were categorized into the following groups:

1. Mineral-Dissolving: This group includes two strains with identical Ms-Cu(I)-ATPases: *Agrobacterium pusense* SX41, which was isolated from tuff in China, and *Aureimonas altamirensis* DSM 2188, which was isolated from Paleolithic wall paintings in the Altamira cave in Spain.
2. Human and Animal Pathogens: This group includes 17 strains isolated from human clinical samples and one potential foodborne strain from a chicken gut. These pathogens are from four families: *Aurantimonadaceae*, *Brucellaceae*, *Stappiaceae*, and *Paracoccaceae* (Rhodobacterales).
3. Extremophiles: This group includes eight strains that were isolated from wastewater and industrial pollutants: *B. anthropi* MWF001, *B. anthropi* SBA 01, *Shinella* sp. HZN7, and *Ochrobactrum* sp. AP1BH01-1, which belong to the *Rhizobiaceae* family; *Pannonibacter phragmitetus* CGMC9175 and *Stappia* sp. PZPMeth1 from the *Stappiaceae* family; and *Pseudochelatococcus contaminans* DSM 28760 and *P. lubricantis* CCM8528 from the *Chelatococcaceae* family.
4. Miscellaneous: The three strains in this category were collected from unrelated or unspecified sources: *B. anthropi* LMG 5140 was isolated from cattle-dip trays in Australia; *B.* sp. NBRC 13694, an airborne strain, does not have any information about its country of origin; and *Ochrobactrum* sp. BTU 2 is an environmental isolate that was collected in Germany.
5. Soil- and Plant-Associated: This category consists of four strains grouped in the *Brucellaceae* family that inhabit soil (*Falsochrobactrum shangaiense* HN4), the rhizosphere, and root nodules (*B. lupini*) isolated from *Lupinus albus* in Argentina.
6. Aquatic and Free-Living: Five strains of *Tristella mobilis* (*Geminicoccales*) were isolated from the Indian Ocean, *Brucella* sp. MAB 22 was collected from a lake in China, and *Filomicrobium* sp. was isolated from estuarine sediment in China.
7. Unknown: One strain did not have any information on its source.

Recent advances in phylogenomics, based on whole-genome comparisons, suggest no significant differences between *Brucella* and *Ochrobactrum* to justify their separation into two distinct genera. Thus, species formerly classified as *Ochrobactrum* have been reclassified as *Brucella* [39]. Beyond these taxonomic differences, it is operationally important to preserve database nomenclature to avoid information loss. In this study, we used NCBI’s Identical Protein Groups nomenclature, which is compatible with the NCBI tools used in this study (Table S1).

### 3.4. Prevalence of Identical Nucleotide Sequences Encoding Ms-Cu(I) ATPase in the 43 Strains

To better understand the evolution of these identical amino acid sequences, and recognizing that the genetic code is degenerate and that identical proteins do not necessarily correspond to identical nucleotide sequences, we examined whether the nucleotide sequences encoding the 43 Ms-Cu(I) ATPases are also identical. We aligned the nucleotide sequences of the 43 Ms-Cu(I) ATPases using Clustal O. The MSA revealed only one mismatch between two strains: *Aureimonas altamirensis* A31 and *Aureimonas* sp. OT7. The mismatch occurred at the third position of one alanine codon in each genome, resulting in a silent mutation that does not affect the amino acid sequence (Figure S2).

### 3.5. The 43 Ms-Cu(I)-ATPase Genes Are Part of a 22-Gene Cluster Predicted to Be Functionally Related to Metal Homeostasis

To investigate the co-occurrence and prevalence of Ms-Cu(I) ATPase genes, we used the NCBI Graphics resource to examine the gene neighborhoods of the 43 nucleotide sequences encoding Ms-Cu(I)-ATPases (*copA* genes). This genome-by-genome manual examination revealed that *copA* genes are part of an extensive, highly conserved syntenic region across the 43 species. Neighborhood analysis of the regions upstream and downstream of the *copA* genes showed that they are bordered by homologous genes encoding proteins predicted to be involved in copper homeostasis (Table 1 and Figure S3). The first neighboring gene encodes a putative multispecies multicopper oxidase (MCO) domain-containing protein (WP_012092678.1), a multidomain enzyme that couples substrate oxidation to dioxygen reduction to water. The MCO CueO of *E. coli* oxidizes toxic Cu(I) to less toxic Cu(II), thereby maintaining copper homeostasis [40]. The identity between the putative MCO and the characterized *E. coli* CueO protein was 35.19% based on Blastp suite-2 sequences, with an E-value of 3 × 10^−11^ and 40% coverage alignment. The second gene in the cluster downstream of *cueO* was annotated as a multispecies cation diffusion facilitator (CDF) family transporter (WP_012092677.1). The CDF family members maintain cytoplasmic metal homeostasis by exchanging protons for metal ions [41]. This arrangement is intriguing, as no reports describe CDF transporters recognizing copper as a substrate. The CD-Search comparison indicates homology with CzcD (89% query coverage, E-value = 1.66 × 10^−119^), which is involved in Co/Zn/Cd efflux. The third gene encodes Ms-Cu(I)-ATPase (*copA*). The fourth gene (WP_012092675.1) encodes a homolog (45% identity, 71% query coverage, and E-value of 6 × 10^−35^) of *Pseudomonas aeruginosa* CopG, an oxidoreductase (formerly classified as DUF411) that is frequently found in clusters of copper resistance genes [42]. These four proteins are included in the IPG database and were consistently found in the 43 genomes analyzed in this study using the NCBI tool Graphics (Table S3) and CAGECAT (Figure 1B). The proteins encoded by the fifth (WP_028034404.1) and seventh (WP_231771508.1) genes do not have recognizable domains and are uncharacterized. The sixth gene (WP_231771507.1) encodes a CopM (*sll0788*) homolog (41% identity, 64% query coverage, and E-value of 2 × 10^−14^) of *Synecocystis* sp. PCC 6803, which is involved in copper resistance and homeostasis [43,44].

**Table 1:** List of proteins encoded in the five gene clusters identified in chromosome 2 of *Brucella anthropi* ATCC 49188. Each color represent a different function assigned is crucial for understanding gene clusters.

Cluster	Protein ID	DNA Strand	Gene Start	Gene End	Product
ARGC	WP_049768451.1	−	521,109	522,305	ArsK, MFS transporter
	WP_012092640.1	−	522,332	523,048	Arsenical resistance protein ArsH
	WP_012092641.1	−	523,045	524,133	ArsB, As-efflux, arsenical-resistance protein
	WP_012092642.1	−	524,161	524,586	Glutaredoxin, arsenate reductase
	WP_012092643.1	−	524,595	525,131	ArsC, arsenate reductase
	WP_012092644.1	−	525,136	525,495	ArsR, transcription factor
	WP_012092645.1	+	525,542	526,039	DUF6428
MTGC	WP_012092646.1	−	526,145	526,609	signal peptidase II
	WP_012092647.1	−	526,635	527,378	cytochrome C biogenesis protein CcdA
	WP_012092648.1	−	527,378	527,998	Sco (synthesis of cytochrome C oxidase) protein
	WP_012092649.1	−	527,995	528,498	Cu chaperone PCuAC
	WP_012092650.1	−	528,491	529,108	CzcD CDF protein
	WP_028034401.1	−	529,175	531,307	ZntA P-type ATPase
	WP_012092652.1	+	531,374	531,790	CadR/Pbr-like protein, MerR family transcription factor
	WP_012092653.1	−	531,798	532,733	bile acid:sodium symporter
	WP_012092654.1	−	532,742	533,107	Ars/SmtB transcription factor
CGC	WP_012092655.1	+	533,536	534,534	P-type conjugative transfer ATPase TrbB
	WP_012092656.1	+	534,531	534,860	conjugal transfer protein TrbC
	WP_012092657.1	+	534,860	535,141	conjugal transfer protein TrbD
	WP_012092658.1	+	535,150	537,660	conjugal transfer protein TrbE
	WP_012092659.1	+	537,657	538,439	conjugal transfer protein TrbJ
	WP_012092660.1	+	538,456	538,743	conjugal transfer protein TrbK
	WP_012092661.1	+	538,747	540,105	conjugal transfer protein TrbL
	WP_012092662.1	+	540,102	540,791	conjugal transfer protein TrbF
	WP_012092663.1	+	540,788	541,816	P-type conjugative transfer protein TrbG
	WP_012092664.1	+	541,813	542,940	conjugal transfer protein TraB/TrbI
MHGC	WP_012092665.1	−	542,943	543,182	hypothetical protein
	WP_012092666.1	−	543,136	544,083	LysR family transcription factor
	WP_012092668.1	−	544,473	544,787	Metal-sensing transcription factor
	WP_040128397.1	−	544,784	545,830	zinc transporter ZitB
	WP_012092670.1	−	545,987	547,492	MFS transporter
	WP_012092671.1	+	547,962	548,177	heavy metal transport/detoxification protein CopZ
	WP_231771508.1	−	548,259	548,717	hypothetical protein
	WP_231771507.1	−	548,831	550,384	CopM homolog metallochaperone
	WP_028034404.1	−	550,503	550,913	hypothetical protein
	WP_012092675.1	−	551,092	551,580	resistance to cations CopG homolog
	**WP_012092676.1**	−	**551,881**	**554,268**	**Cu (I) ATPAse 1**
	WP_012092677.1	−	554,374	555,339	Co/Zn/Cd CDF protein
	WP_012092678.1	−	555,409	556,917	multicopper oxidase, CueO
	WP_197057541.1	−	556,991	557,350	hypothetical protein
RHGC	WP_244471115.1	+	557,466	557,927	cytochrome c
MHGC	WP_028034408.1	−	558,034	558,240	hypothetical protein
	WP_012092680.1	−	558,378	558,758	transcription factor CueR
RHGC	WP_012092681.1	−	558,779	559,795	oxidoreductase iron-sulfur binding domain
	WP_012092682.1	−	559,807	560,070	NrdH-redoxin
MHGC	WP_012092683.1	−	560,123	562,681	Cu (I) ATPAse 2
	WP_012092684.1	+	562,841	563,035	heavy metal transport/detoxification protein
	WP_012092685.1	+	563,050	563,454	hypothetical protein
RHGC	WP_028034411.1	+	563,914	564,189	CsoR-like protein
	WP_012092687.1	−	564,208	564,975	glutaredoxin family protein
	WP_012092688.1	+	564,998	565,501	DUF305 domain-containing protein
	WP_012092689.1	−	565,633	566,433	TlpA, redoxin domain-containing protein
	WP_012092690.1	−	566,435	568,243	DsbB, thioredoxin-domain protein
	WP_040128738.1	−	568,285	568,893	DsbA, oxidoreductase
	WP_012092692.1	−	569,080	569,619	thiol:disulfide interchange protein, thioredoxin family

**Figure 1:**
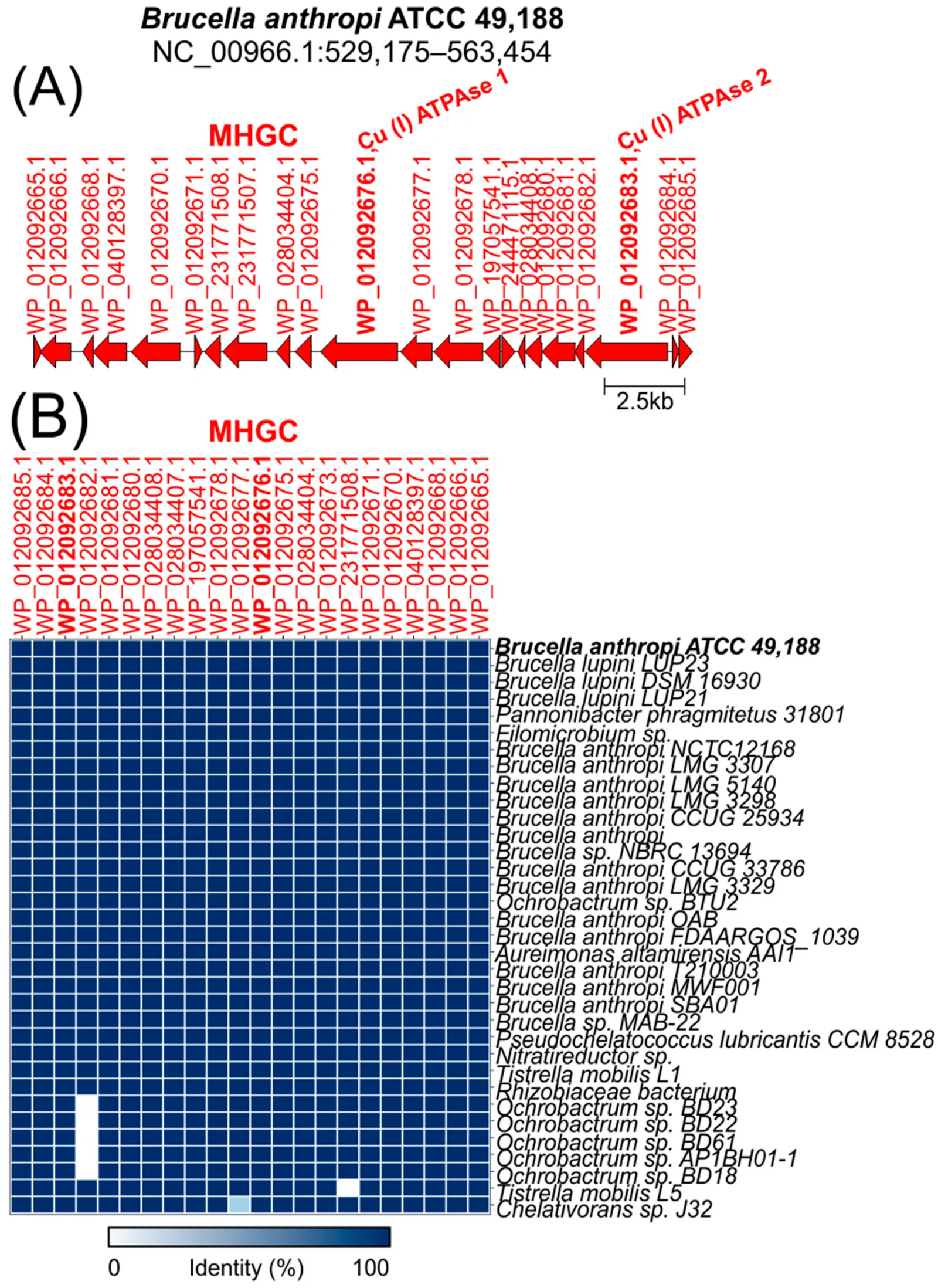
The Ms-HMT-P-type ATPase (WP_012092676.1) is located in an MHGC. (**A**) The analysis of gene context and synteny analysis reveal WP_012092676.1 is conserved in distantly related Alphaproteobacteria as analyzed by CAGECAT-clinker. (**B**) Protein sequence conservation across multiple alphaproteobacterial genomes according to CAGECAT-cblaster. The protein IDs (RefSeq) encoded are indicated for each gene using the *B. anthropi* ATCC 49188 as a reference.

The Gene Neighborhoods (GNs) upstream and downstream of *copA* contain 22 genes that are functionally related to metal transport, 12 of which encode proteins potentially involved in maintaining Cu(I) homeostasis. Based on these predictions, we named the genetic region the Multimetal Homeostasis Gene Cluster (MHGC). Table 1 (pink-shaded region) lists the 22 proteins in the GN of the *copA* gene of *B. anthropi* ATCC 49188, which were found in all 43 genomes (Table S3). A homolog of Ms-Cu(I)-ATPase (52% identity, 83% query coverage, and E-value of 0.0) was found in this GN. To avoid confusion, they were designated Ms-Cu(I)-ATPase1 (WP_012092676.1) and Ms-Cu(I)-ATPase2 (WP_012092683.1). Both proteins share the same conserved domains (according to CD-Search). The colocalized genes in these genomic regions are believed to function together to confer metal tolerance. The Gene Neighborhood (GN) analysis of 30 genes upstream and 30 genes downstream of the *copA* gene across the 43 strains allowed us to identify shifts in predicted protein function and changes in gene orientation, which indicate the end of a cluster and the beginning of a new cluster, helping us define the extent of this MHGC and its boundary genes. We inferred collinearity among the MHGC and its bordering clusters, the Redox Homeostasis Gene Cluster (RHGC) and Conjugation Gene Cluster (CGC), which are described in the following sections. The boundary between the MHGC and CGC was easy to define because of shifts in protein function and gene orientation. The separation between the RHGC and MHGC was difficult to define based on different protein functions due to the presence of three proteins predicted to participate in redox homeostasis that were encoded together or close to Cu(I)-ATPase1 and Cu(I)-ATPase2 (Table 1, magenta-shaded region). We separated the two clusters using the weak criterion of the absence of a transporter as the RHGC lacks metal transporters. The co-localization of redox and metal homeostasis genes is poorly understood.

To continue characterizing the GN of the MHGC, we selected the genome of the type strain *B. anthropi* ATCC 49188 as a synteny model for the 43 genomes.

### 3.6. Description of the Redox Homeostasis Gene Cluster (RHGC) Encoded Upstream of MHGC

Within the RHGC, two putative glutaredoxins and three thioredoxin proteins with predicted disulfide oxidoreductase activity may catalyze the disulfide bond formation required for proper protein folding. In addition to this function, glutaredoxins and thioredoxins, the major antioxidant enzymes in bacteria, help reduce reactive oxygen species (ROS) levels under stress conditions induced by heavy metals, antibiotics, and xenobiotics [45].

According to CAGECAT, this collinearity between the MHGC and RHGC is present not only in *B. anthropi* ATCC 49188 (Figure 2A), but also in 28 other strains (Figure 2B, Table 1, magenta-shaded region).

### 3.7. A Cluster of 10 Genes Encoding Proteins for the Type IV Secretory Pathway, Putatively Involved in Conjugative DNA Transfer, Is Located Downstream of the MHGC

Gene context analysis revealed a cluster of 10 *trb* genes (*trbBCDEJKLFGI*) downstream of the MHGC (Figure 3A)—also known as *tra* or *vir* genes in other bacteria—are located on the complementary strand. The short distances between the genes within the cluster (Table 1) suggest they likely form an operon. The products of these genes are predicted to participate in various steps of conjugative DNA transfer. We named this multispecies cluster the Conjugation Gene Cluster (CGC), and it is collinear with the MHGC and RHGC in at least 24 of the bacterial strains analyzed (Figure 3B). The major synteny break in this CGC was the absence of the entire CGC in *Shinella* sp. HZN7, which was replaced by a 25 kb gene cluster encoding transposases, relaxases, methylases, a toxin–antitoxin stability system, endonucleases, and integrases, resembling components of mobile genetic elements (MGEs) (Figure S4).

### 3.8. Co-Occurrence of a Miscellaneous Transporter Gene Cluster and an Operon Encoding a Cytochrome C Assembly System

At 200 base pairs downstream of the TrbB-encoding gene on the opposite DNA strand, we found a gene encoding a protein annotated as a metalloregulatory transcriptional repressor belonging to the ArsR/SmtB family (WP_012092654.1) (Figure 4 and Table 1). This gene appears to form an operon with a gene encoding a predicted bile acid sodium symporter (BASS, WP_012092653.1), which is grouped in the sodium/solute symporter family and is widely distributed across prokaryotic and eukaryotic genomes. In the mammalian liver, BASS participates in Na^+^–taurocholate cotransport; in plants, it can influence salt tolerance; however, in bacteria, its role remains unknown [46]. Downstream of this probable operon on the opposite strand, we found a Helix–Turn–Helix CadR/PbrR-like transcription regulator (WP_012092652.1), which may regulate the expression of the upstream zinc/cadmium/lead P-type ATPase (WP_028034401.1) [47]. Seventy nucleotides downstream of this P-type ATPase is a putative operon involved in the biogenesis of cytochrome c oxidase (WP_012092650.1 and WP_012092646.1). The first gene in this likely operon encodes a homolog of CzcD, a CDF-family protein required for the active efflux of divalent cations, including cobalt, zinc, and cadmium [41]. The stop codon (TGA) of *czcD* overlaps the start codon (ATG) of the adjacent gene that encodes a potential PCu(A)C, a periplasmic Cu chaperone involved in the assembly of cytochrome c oxidase [48]. The SCO and CcdA proteins are required for cytochrome c oxidase synthesis [49,50]. The last gene encodes signal peptidase II, which may be involved in secreting cytochrome c oxidase to the periplasmic side of the membrane. We named this group of genes the miscellaneous transporter gene cluster (MTGC), as it encodes various metal transporters, as well as cytochrome c biosynthesis and regulatory proteins (Figure 4A). This MTGC is present alongside the MHGC, RHGC, and CGC in at least 24 of the bacterial strains analyzed (Figure 4B and Table 1).

**Figure 2:**
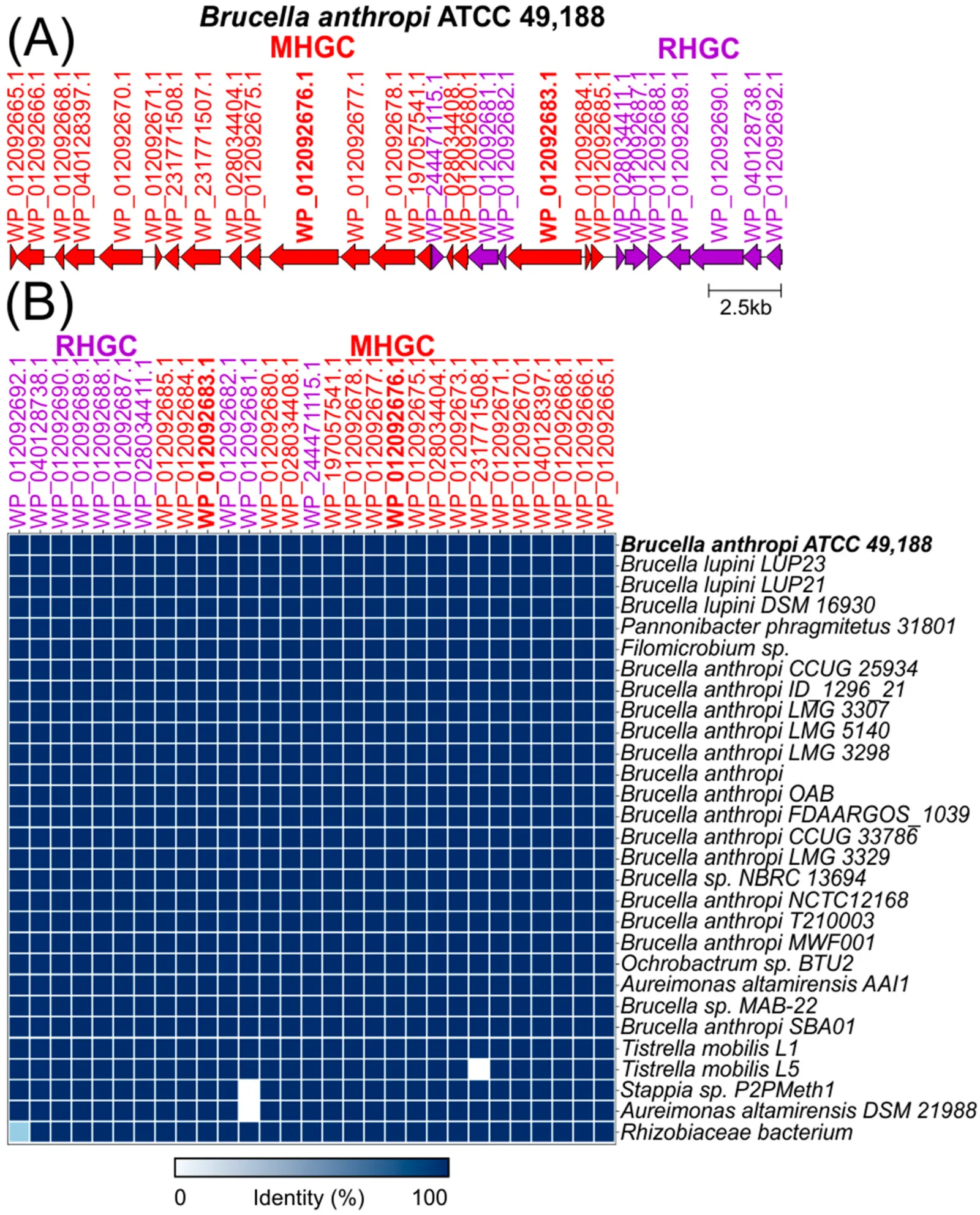
Gene context of the MHGC reveals a redox homeostasis cluster (RHGC). (**A**) The analysis of gene context and synteny analysis reveal the RHGC is conserved in distantly related Alphaproteobacteria as analyzed by CAGECAT-clinker. (**B**) Protein sequence conservation across multiple alphaproteobacterial genomes according to CAGECAT-cblaster. Encoded protein IDs (RefSeq) and annotation are indicated for each gene using the *B. anthropi* ATCC 49188 as a reference, red bold indicates the position of Cu (I) ATPase 1 and Cu (I) ATPase 2 as indicated in Table 1.

**Figure 3:**
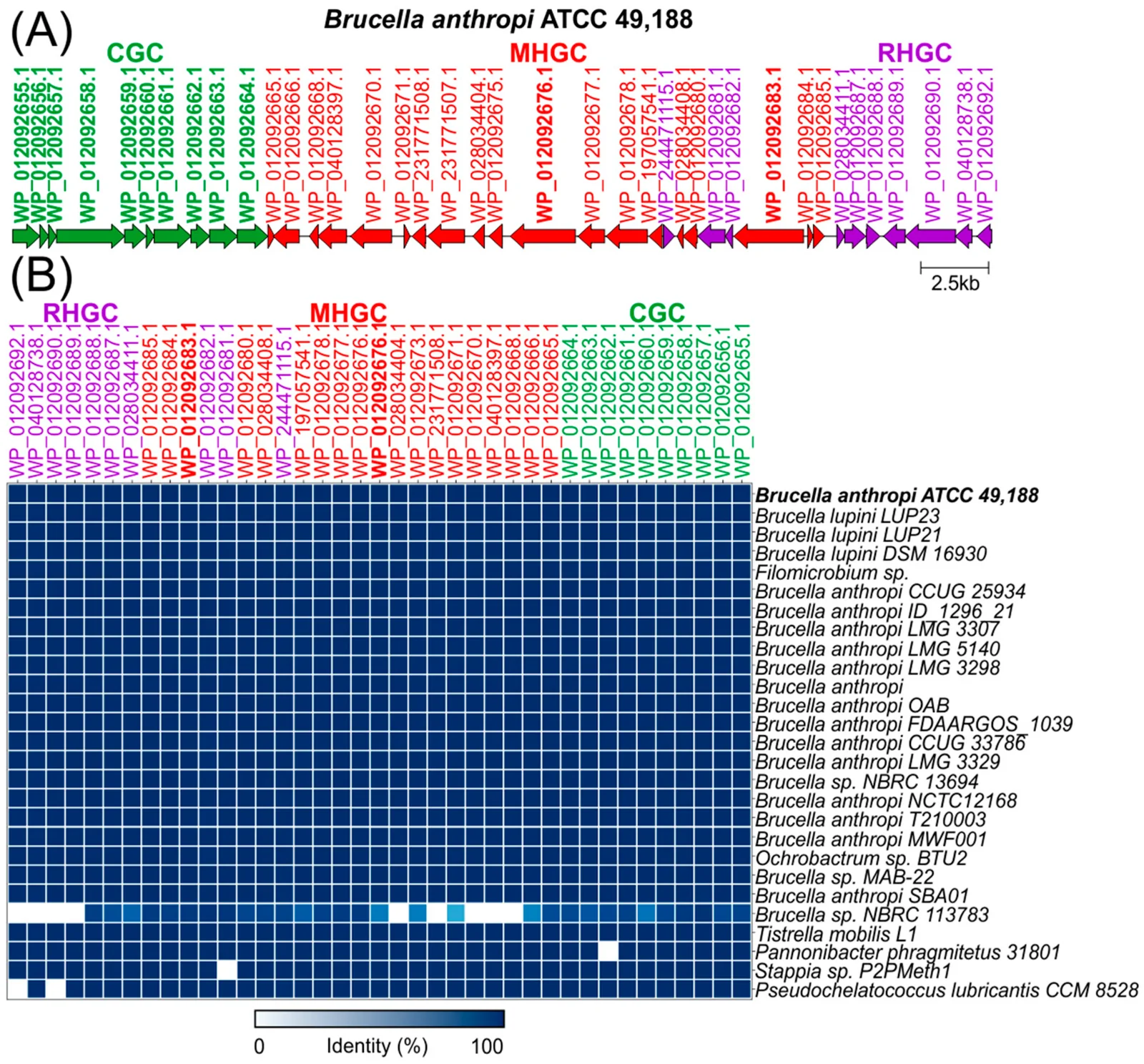
Gene context of the MHGC reveals a conjugation gene cluster (CGC). (**A**) The analysis of gene context and synteny analysis reveal the CGC is conserved in distantly related Alphaproteobacteria as analyzed by CAGECAT-clinker. Red bold indicates the position of Cu (I) ATPase 1 and Cu (I) ATPase 2 as indicated in Table 1. (**B**) Protein sequence conservation across multiple alphaproteobacterial genomes according to CAGECAT-cblaster. Encoded protein IDs (RefSeq) and annotation are indicated for each gene using the *B. anthropi* ATCC 49188 as a reference.

### 3.9. A Putative Operon Encoding an Arsenic-Detoxifying System Lies Downstream of the MTGC

Arsenic is a highly toxic metalloid that is widespread in the environment. The bacterial resistance operons *arsRBC* and *arsRDABC* are the main detoxification systems [51].

Upstream of the signal peptidase II and the DUF6428 protein mentioned above on the complementary strand is a putative operon involved in arsenic detoxification. The first gene encodes an ArsR-family transcriptional regulator. Downstream of ArsR, two arsenate reductases are encoded, i.e., one from the LMWP family and the other from the thioredoxin-like family, both of which catalyze the reduction of arsenate to arsenite. Arsenite efflux from the cell can be mediated by two transporters encoded downstream of *arsC* and *arsB*, one of which is the arsenite MFS transporter (Figure 5A and Table 1). We named this multispecies cluster the Arsenic Resistance Gene Cluster (ARGC), and it is collinear with the other four previously described clusters—MTGC, CGC, MHGC, and RHGC—in our reference genome (*B. anthropi* ATCC 49188) (Figure 5 and Table 1).

**Figure 4:**
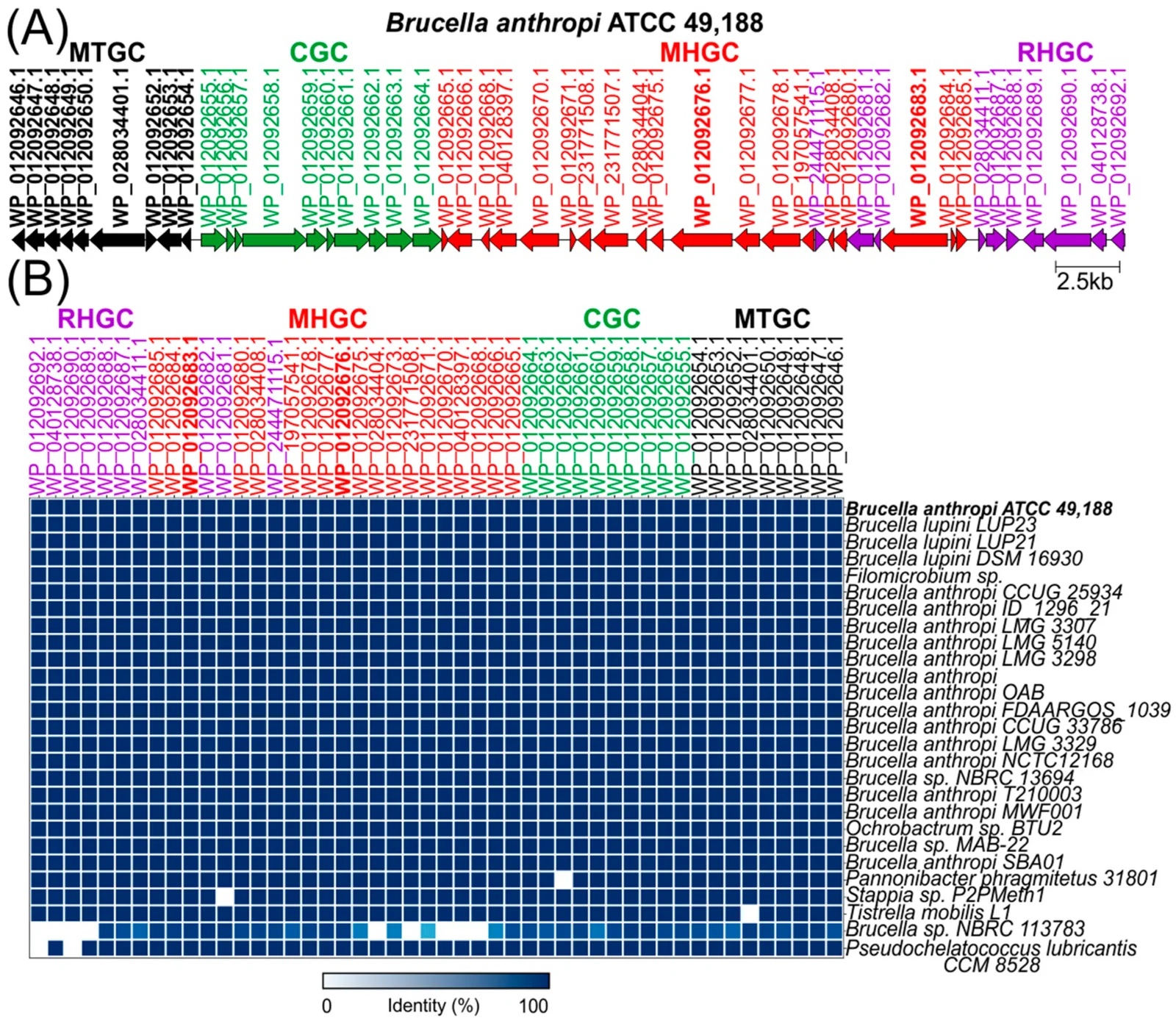
Gene context of the MHGC reveals a second cluster involved in metal transport that we called the miscellaneous transporter gene cluster (MTGC). Red bold indicates the position of Cu (I) ATPase 1 and Cu (I) ATPase 2 as indicated in Table 1. (**A**) The analysis of gene context and synteny analysis reveal the CGC is conserved in distantly related Alphaproteobacteria as analyzed by CAGECAT-clinker. (**B**) Protein sequence conservation across multiple alphaproteobacterial genomes according to CAGECAT-cblaster. Encoded protein IDs (RefSeq) and annotation are indicated for each gene using the *B. anthropi* ATCC 49188 as a reference.

We found that this arrangement of five collinear clusters is conserved in 29 strains according to the CAGECAT analysis [52]. The five clusters span a 48.5 kb region on chromosome 2 in *B. anthropi* ATCC 49188 (Figure 5B).

### 3.10. The Five Collinear Gene Clusters of B. Anthropi ATCC 49188 Are Conserved in Distantly Related Alphaproteobacteria

Synteny is the conservation of gene order across species over evolutionary time [53]. We observed that the 48.5 kb region encompassing the five collinear clusters is highly conserved across 29 strains (Figure 5B). Based on functional inference from similarity, putative operonic structure, gene orientation, and conserved domains, we defined five gene clusters. Our analysis using CAGECAT-cblaster revealed the presence or absence of homologs for each gene; however, it did not provide gene directionality. As described in the methods, we used CAGECAT-clinker to characterize the configuration of the gene clusters in terms of directionality, the strand on which each gene is encoded, and the distances between genes within a given cluster. This approach allowed us to investigate the gene context of the *B. anthropi* ATCC 49188 clusters and determine synteny across other strains and species. We discovered that the five collinear clusters are also conserved outside the genus *Brucella* in distantly related species, namely *Filomicrobium* sp. (Rhizobiales-Hyphomicrobiaceae), *Stappia* sp. P2PMeth1 (Rhizobiales-Stappiaceae), and *Tistrella mobilis* (Geminicoccales-Geminicoccaceae) (Figure 6), suggesting that these five collinear clusters were transferred or mobilized. We named this genomic arrangement the Collinear Synteny Block (CSB) based on the collinearity of the five gene clusters, their highly conserved synteny, and their predicted interrelated function [54].

**Figure 5:**
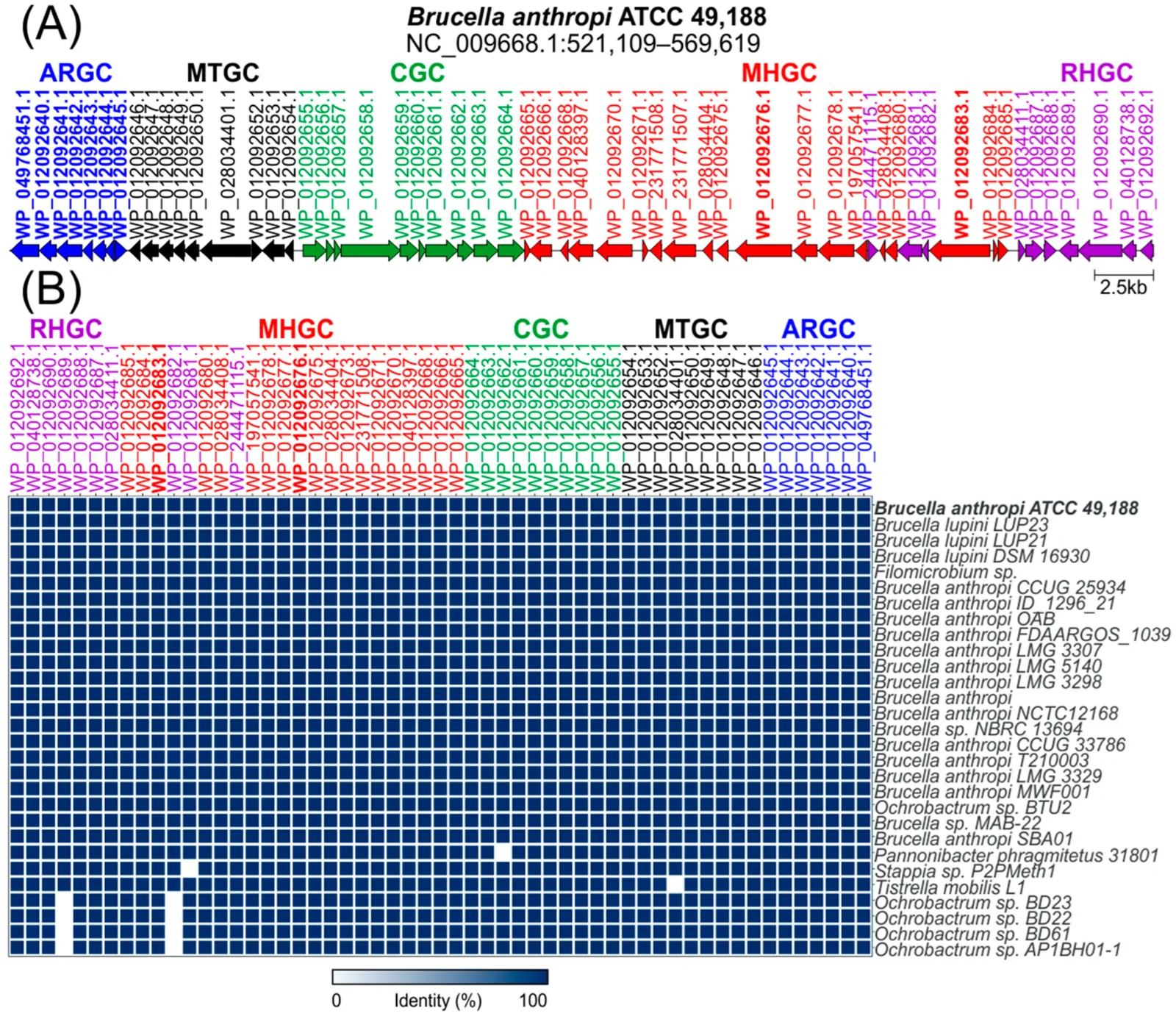
Discovery of a ~48.5 kb colinear syntenic block containing 5 clusters. (**A**) The gene context of the MHGC reveals the presence of an arsenic resistance cluster (ARGC). Red bold indicates the position of Cu (I) ATPase 1 and Cu (I) ATPase 2 as indicated in Table 1. (**B**) Protein sequence conservation across multiple alphaproteobacterial genomes according to CAGECAT-cblaster. Encoded protein IDs (RefSeq) and annotation are indicated for each gene using the *B. anthropi* ATCC 49188 as a reference. These results have an accompanying table (Table S2) and both indicate the organization and order of the five clusters relative to one another. We excluded *Aureimonas altamirensis A31* and *Pseudochelatococcus contaminans DSM 28760* from the best-score list because they lack the required RHGC due to the end of the contig. *Paracoccus yeei* FDAARGOS_643 was also excluded due to the absence of the ARGC. *Shinella* sp. HZN7 was not included in the best score table because this strain lacks CGC, MTGC, and ARGC (Figure S5).

**Figure 6:**
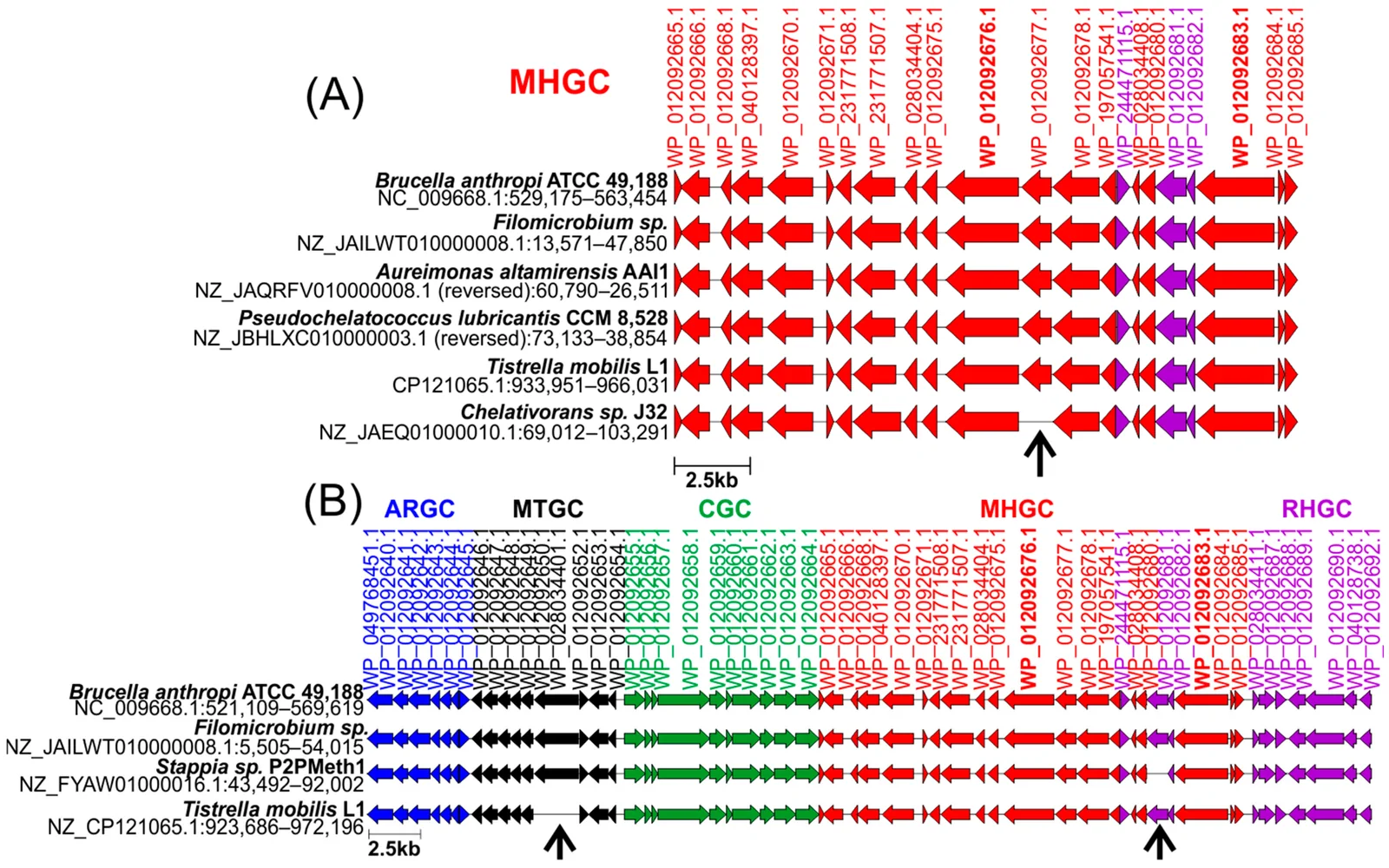
Distribution of the MHGC and the CSB across families of Alphaproteobacteria. (**A**) The arrangement of the MHGC is highly conserved in species from evolutionary distant Rhizobiales, Geminicoccales, and Rhodobacterales orders (see also Figure S5). (**B**) The arrangement of the CSB is highly conserved across alphaproteobacterial species. The missing genes in the cluster relative to the *B. anthropi* ATCC 49188 genome are indicated with black arrows.

### 3.11. The CSBs from Different Species Are Located in Divergent Genomic Islands

Integrative and conjugative elements (ICEs) are self-transmissible, mobile genetic elements integrated into the bacterial chromosome and play a key role in microbial evolution [55]. We analyzed the gene context of the CSBs to determine whether they belonged to a larger ICE, such as a GI. We used the IslandViewer4 web server to search for precomputed GI predictions in the genomes of *B. anthropi* ATCC 49188 (listed as *Ochrobactrum anthropi* ATCC 49188 in the IslandViewer4 database), *O. anthropi* OAB, and *Brucella* sp. MAB-22. A potential GI (109.24 kb) containing the 48.5 kb CSB in the three analyzed genomes was predicted by IslandViewer4 and was designated Genomic Island *Brucella anthropi* (GIBan) (Figure S5). Figure S6 is a screenshot of the IslandViewer4 output. Figure S6A shows a nucleotide composition bias in the inner circle that fits with the predicted GI on chromosome 2, as detected by the IslandPath-DIMOB predictor (continuous blue line). Additionally, the SIGI_HMM predictor identified multiple codon usage biases overlapping the same region on chromosome 2 (discontinuous orange lines). Figure S6B shows a table of the genes and their encoded proteins that are predicted to be located within regions of dinucleotide sequence bias and/or codon usage bias (blue and yellow lines, respectively). We downloaded this protein list and updated the annotations using the NIH Protein database, the Conserved Domains (NCBI), and literature analysis (Table S2). This analysis led us to identify a cluster of mercury resistance proteins, constituted by a homolog of the MerR transcription factor family (WP_012092599.1), a mercury transporter (MerT) homolog (WP_012092600.1), and a mercury reductase MerBA homolog (WP_012092603.1). This cluster was located 35.5 kb away from the CSB (Table S2).

Figure S6C shows a broader view of the predicted 109.24 GI (blue and orange lines), the genes encompassed by the island, and their locations on the (+) (pink) or (–) (green) DNA strand. We also added the locations of the CSB encompassing the five gene clusters for clarity. We also identified the potential recombinase that mediates GIBan integration. According to the NCBI Graphics resource, the genes encoding the recombinase and tRNA-Met are adjacent, encoded on opposite strands, and overlap by 40 nucleotides, as shown in Figure S7. DR1 is 18 nucleotides long (ACC AAC GCC CCC GTC CTA) and is located at the recombinase–tRNA overlap site (Figure S7). Figure 7 shows a genetic map based on the GIBan that accurately integrates the positions of the CSB and GIBan, including the genes encoding the recombinase, the tRNA-Met integration sites, and the resulting DRs. IslandViewer4 predicts the GIBan length to be 109.24 kb, but it does not include the recombinase and tRNA. Including the integration sites and the recombinase, the estimated GIBan size is 111.1 kb (Figure 7A).

Table S3 also shows that several species lacked a recombinase-family protein, a tRNA that could serve as an integration site, and DRs. In the genomes of three *Aureimonas* strains and one *Agrobacterium* strain, the CSBs are not located within GIs (Table S3 and Figure S6D).

### 3.12. Co-Occurrence of the CSB and Potential Multidrug Resistance Proteins Encoded on Chromosome 2 of B. anthropi ATCC 49188

Numerous studies have reported the co-occurrence of antibiotic- and metal-resistance genes (see [56] for a review). To search for this co-occurrence on chromosome 2 where the CSB is located, we used the search-a-gene facility in the IslanderView4 toolbox to identify putative multidrug efflux proteins. A search for the abbreviations MFS and RND and the word resistance retrieved 20, four, and two putative transporters, respectively. We compared their annotated functions and predicted substrates with the Comprehensive Antibiotic Resistance Database (CARD) using standard BLAST, which was implemented in this bioinformatics resource [36]. Table S4 shows homologs with a potential role in antibiotic resistance (yellow-shaded proteins). All these putative efflux pumps were found to be scattered on chromosome 2 of *B. anthropi* ATCC 49188.

## 4. Discussion

### 4.1. The Co-Occurrence of Multimetal Homeostasis Gene Clusters in Rhizobiales

In this study, we identified a more complex genomic organization of the MHGC in the human pathogen *B. anthropi* ATCC 49188 (Rhizobiales) than that reported for other MHGCs [20] (Dávalos and García-de los Santos, 2023). It involves identical proteins that, according to the IPG database (NCBI), share the same RefSeq, nucleotide, and amino acid sequences and lengths. The identical Ms-Cu(I)-P-type-ATPase1 (RefSeq WP_012092676.1, CopA1) was found in the genomes of 43 strains isolated from different environments and countries, which span across three orders within the class Alphaproteobacteria: Rhizobiales, Geminicoccales, and Rhodobacterales. The neighborhood analysis of Ms-Cu(I)-ATPase1 revealed a second Ms-Cu(I) ATPase, CopA2 (WP_012092683.1). Both CopA1 and CopA2 are encoded within the same MHGC of 21 collinear and syntenic genes, most of which may be operons involved in maintaining metal homeostasis. The MHGC found in *B. anthropi* ATCC 49188 lacks the CusABC system and the Ni/Co resistance system RcnA/R reported by [20] Dávalos & García-de los Santos (2023) for *Methylobacterium extorquens*. Instead, it encodes at least two CDF proteins that might confer Co/Zn/Cd tolerance and two Cu(I)-P-type ATPases. Of the 43 genomes analyzed, 21 belong to the *Brucellaceae* family in the order Rhizobiales. We selected the MHGC of *B. anthropi* ATCC 49188 as a representative model throughout the study.

### 4.2. The Five Collinear Gene Clusters May Contribute to Antimicrobial Resistance

We thoroughly analyzed the proteins encoded by the five gene clusters to determine their biological roles. The separation between the RHGC and MHGC was difficult to define. We distinguished the RHGC from MHGC primarily based on the prevalence of redox-active proteins and the absence of copper efflux transporters. By contrast, the MHGC encodes 22 proteins; 19 are associated with metal transport, and most of them are encoded in the same orientation. Copper-mediated oxidative stress is primarily controlled by the thiol-rich buffering system, copper chaperones, copper-efflux systems, and transcriptional regulators. At the genomic level, this is reflected in the synteny of genes encoding these functions. This functional interplay makes separation difficult and suggests that the RHGC and MHGC may constitute a single gene cluster for redox and metal homeostasis. Only three proteins could be involved in redox homeostasis. The RHGC encodes two glutaredoxin-like proteins and three putative thioredoxin proteins, both predicted to have disulfide oxidoreductase activity. They may catalyze disulfide bond formation required for proper protein folding. In addition to this function, glutaredoxins and thioredoxins, the major antioxidant enzymes in bacteria, help reduce ROS levels under stress conditions. The most important source of ROS for intracellular pathogens is macrophages, the specialized cells of the innate immune system that kill invasive microorganisms [57]. Bacteria exposed to antibiotics, heavy metals, and xenobiotics also experience oxidative stress [45,58,59]. It is well documented that ROS participate in the decomposition of organic matter in various environmental settings, including soil, sediments, water, and the atmosphere [60]. Adjacent to the RHGC are numerous proteins involved in metal resistance (Cu, Zn, Co, and Cd resistance). These genes may allow bacteria to adapt and survive in metal-rich environments. The potential involvement of these MHGCs in metal resistance correlates with the metal-rich habitat reported for most of the species analyzed in this study. Notably, genes encoding a C-type cytochrome, a 2Fe-2S cluster domain protein, and a glutaredoxin protein are located near genes encoding Cu(I)-ATPases, even in phylogenetically distant species. Delimiting the RHGC and MHGC based on predicted protein functions was unclear because three redox-active proteins (cytochrome c, an oxidoreductase 2Fe-2S-binding protein, and NrdH-redoxin) are encoded between Ms-Cu(I)-ATPase1 and Ms-Cu(I)-ATPase2 (Table 1). The genes encoding 2Fe-2S-binding protein and NrdH-redoxin are together and syntenic in the 43 genomes analyzed.

The conjugation gene cluster (CGC) is presumed to participate in pilus formation and the DNA transfer required for CSB dispersion. Additional TraA (WP_012090908.1), TraC (WP_012090907.1), TraD (WP_012090906.1), and TraG (WP_012090905.1) proteins are encoded on chromosome 1 of *B. anthropi* ATCC 49188. To determine whether the conjugation gene cluster (CGC) in the CSB contains all components required for conjugative transfer, we compared it with the conjugative transfer system encoded by the plasmid pTiC58 from *Agrobacterium tumefaciens*, which is taxonomically close to *Brucellaceae* (order Rhizobiales). We noted that homologs of the VirA, VirC, VirD, and VirG proteins required for DNA processing are absent from the CGC and from chromosome 2 of *B. anthropi* 49188 but are present on chromosome 1, which may work together. Comparison of the trb operons revealed the absence of *trbH* in *B. anthropi* ATCC 49188. Deletion of *trbH* in *A. tumefaciens* significantly reduces the transfer frequency of plasmid pTiC58, but it is not indispensable for conjugation [61]. Determining the role of the CGC in CSB mobilization requires experimental investigation.

The co-occurrence of this cluster of conjugation genes with ARGC may not be random. Numerous studies have demonstrated that metals such as Ag, Cu, Hg, Cr, and Zn stimulate conjugation [62,63,64,65]. Environmental stressors such as ethanol, SDS, iodine, nitrate, nitrite, hydrogen peroxide, salinity, NaOH, and HCl increase the ability of the marine fish pathogen bacterium *Vibrio harveyii* to receive plasmids via conjugation with *E. coli* as the donor [66,67]. In this context, the co-occurrence of antimicrobial resistance genes and conjugation gene clusters may confer an adaptive advantage in maintaining their propagation under environmental stress.

The MTGC is important for two reasons. On the one hand, it may enhance resistance to excessive metal exposure. ZntA is a P-type ATPase that confers resistance to Pb, Zn, and Cd, and CzcD, a CDF family member, effluxes Co, Zn, and Cd. Their putative distinct regulators, ArsR/SmtB and MerR, are adjacent in the genome and can be activated under different environmental conditions. The function of the predicted bile acid sodium symporter (BASS, WP_012092653.1) remains to be elucidated. In contrast, the MTGC and ARGC were separated into two distinct clusters based on the presence of the protein DUF6428 (WP_012092646.1), which has an unknown function and is divergently oriented. According to CDART Search (NCBI), this DUF6428 domain is present in organoarsenical lyase (ArsI), an enzyme that detoxifies methylarsenite [68], and in arsenic resistance N-acetyl transferase (ArsN) [69]. This finding supports the hypothesis that DUF6428 is part of the collinear ARGC. Since the electron acceptor for arsenite oxidase ArsC (WP_012092643.1) has been shown to be a c-type cytochrome [70], we hypothesize that the cytochrome c biogenesis operon located in the MTGC may be part of the ARGC.

### 4.3. Dispersion and Divergence of CSBs

Assuming that CSBs may confer resistance to multiple metals in phylogenetically distant species and that their presence may confer an adaptive advantage in colonizing metal-rich environments, we used IslandViewer4 to predict whether CSBs are contained within a genomic island. This analysis, supported by the IslandPath-DIMOB and SIGI_HMM methods, showed that CSBs were predominantly located within genomic islands >100 kb in size in most of the *B. anthropi* strains analyzed. This prediction was reinforced by identifying the integrase, tRNA-Met integration site, and identical 18 bp direct repeats flanking it. IslandViewer4 also revealed that, in some strains, only one method predicted the GIs, whereas in others, the CSBs were not contained within a GI (Table S3 and Figure S6D). Moreover, we did not detect the integrase, integration sites, or direct repeats. In *Aureimonas altamirensis* strains and *Agrobacterium pusence*, the CSB was located near a GI but not within it (Table S3 and Figure S6D). This apparent divergence may reflect genome rearrangements maintained by the presence or absence of selective pressure, and compositional amelioration makes the GI DNA resemble that of the new genome. These data may help us understand not only the dispersion of CSBs but also their evolution in different bacteria under different environmental conditions [71].

### 4.4. The Presence of CSBs in Bacteria Isolated from Extreme Environments May Indicate Exposure to Metal Stress in Their Niche

Most of the genomes analyzed in this study belong to bacteria inhabiting extreme environments. The ecological conditions of these niches may impose persistent selective pressure, primarily on bacteria classified as human and animal pathogens, as well as on extremophiles and soil-inhabiting bacteria. *Brucella anthropi* strains isolated from clinical samples are intracellular opportunistic pathogens of vertebrates that must contend with metal intoxication imposed by the host through nutritional immunity [72]. Cu(I) is a potent bactericide that accumulates in macrophage phagolysosomes in response to bacterial infection. *B. anthropi* LMG5140 was isolated from a cattle dip tank on a farm in Australia. This is consistent with the practice of adding Cu to livestock feed to enhance the animals’ health and productivity. CSBs were found in numerous strains isolated from industrial waste and wastewater treatment systems, where metal accumulation is common. The *B. lupini* strains are nitrogen-fixing intracellular symbionts that were isolated from the root nodules of leguminous plants; the source of metal-selective pressure in this environment is unknown.

The co-occurrence of antibiotic and metal resistance genes has been well documented [73]. In our study, we identified putative multimetal resistance genes grouped in a CSB, whereas putative antimicrobial resistance genes were scattered across chromosome 2 of *B. anthropi*; this may indicate that different selective pressures are present in different bacterial niches. The uncontrolled use of antibiotics and metals in human medicine and in livestock and poultry, and of pesticides and fertilizers in agricultural fields, ultimately results in their co-occurrence in wastewater treatment systems and freshwater ecosystems and eventually the co-occurrence of their resistance genes [74].

### 4.5. Contribution of Microbial Genome Mining to Our Knowledge of Eco-Evolutionary Genomics

Bacterial genomes are continuously shaped and restructured by genomic rearrangements and HGT. By studying gene organization within a genome, analyzing gene neighborhoods, and compiling information about niches, we can enhance our understanding of how resistomes evolve and disperse across bacterial species in changing environments [75]. Our genomic study contributes to a broader understanding of gene organization, gene-function prediction, and the interactions among metal and antimicrobial resistance genes [76,77].

### 4.6. Limitations of This Study

This study relied primarily on publicly available genomic sequences and annotations; functional assignments were largely computational; genomic-island boundaries were predicted; no experimental gene expression or resistance assays were conducted; and horizontal transfer or conjugative mobility of the proposed CSB was not directly demonstrated.

## Figures and Tables

**Figure 7:**
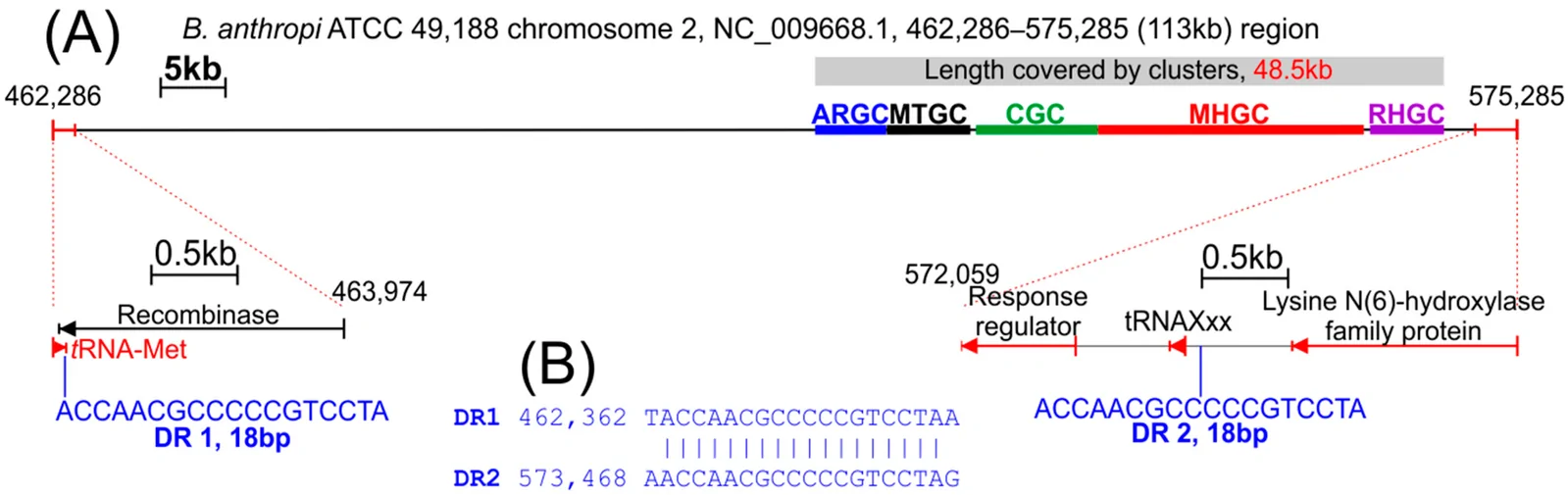
Genetic map of the putative GI on *B. anthropi* ATCC 49188 chromosome 2, as predicted by lslandViewer4. (**A**) The presence of a recombinase-integrase (black arrow, left) on one end, and the presence of two direct repeats, DR1 near a tRNA_Met_, and DR2 near a tRNAXxx on the other end, allowed us to infer the existence of a putative GI. These results are also supported by IslandViewer results, as indicated in Figure S6. (**B**) The distance between the two 18-nt-long DR1 and DR2 is 111.1 kb.

## Data Availability

The original contributions presented in this study are included in the article/Supplementary Materials. Further inquiries can be directed to the corresponding author.

## Supplementary Materials

The following supporting information can be downloaded at https://www.mdpi.com/article/10.3390/microorganisms14102390/s1. Figure S1. Multiple amino acid sequence alignment of Ms-Cu(I)ATPase. Figure S2. Identification of point mutations in the genes encoding the Ms-Cu(I)-ATPase. Figure S3. Screenshot of the gene context. Figure S4. Loss of collinear organization of MHGC and CGC. Figure S5. Multiple *Brucella* strains have putative ~110 kb GIs. Figure S6. A screenshot of the IslandViewer4 output. Figure S7. *Brucella anthropi* ATCC 49188 chromosome 2, complete sequence. Table S1. List of the 43 genomes encoding identical Ms-HMT-P-type ATPase. Table S2. *Brucella anthropi* ATCC 49188 GI protein list. Table S3. Presence/absence of CSB and GI in the 43 genomes. Table S4. Similarity comparison of RND and MFS proteins from *B. anthropi* ATCC 49188 with CARD. Table S5. Software versions, parameters, and access dates.
