# Supplementary material for: Genome-Based Inference of a 50-Kilobase Collinear Synteny Block Containing Genes Predicted to Confer Antimicrobial Resistance and Conjugation Is Widespread Across a Putative Genomic Island in Alpha-Proteobacteria
Source: Microorganisms. 2026 Oct 19;14(10):2390. doi: 10.3390/microorganisms14102390 (PMC13707694; doi:10.3390/microorganisms14102390)

```
                                       10        20        30        40        50        60        70        80        90       100
                              ....|....|....|....|....|....|....|....|....|....|....|....|....|....|....|....|....|....|....|....|
CopA_Calbicans                MQKFIINFANIHCDKCEGIIRNVVSRYYKISDETDLNANIKPSEVIFKLENNEVTLYGNDLIDFHGDIKSIIKSLGKSGFDILSWDLHNGDETVSSSQIV
ATP7b_Dmelanogaster           ----------------------------------------------------------------------------------------------------
CopA_Hpylori                  ----------------------------------------------------------------------------------------------------
CopA_like_Nvulgaris           ----------------------------------------------------------------------------------------------------
CopA_Lpneumophila             ----------------------------------------------------------------------------------------------------
WP_012092676.1_Rhizobiales    ----------------------------------------------------------------------------------------------------

                                      110       120       130       140       150       160       170       180       190       200
                              ....|....|....|....|....|....|....|....|....|....|....|....|....|....|....|....|....|....|....|....|
CopA_Calbicans                NTNIQGANEPDGFIDIVGMYHKFINSRNKKKHMKYCKVCQESQQHKKKSTDSDFSSSSEDTVVEIPHQEFRASFLVTGMTCASCAQSITDNLRSILGESK
ATP7b_Dmelanogaster           ----------------------------------------------------------MPSDERVEATMSTVRLPIVGMTCQSCVRNITEHIGQKSGILG
CopA_Hpylori                  ---------------------------------------------------------------------MKESFYIEGMTCTACSSG-------------
CopA_like_Nvulgaris           ------------------------------------------------------------MDSQTDSVRHRSRMPVRGMTCAVCAGRIEKMVGRMEGVET
CopA_Lpneumophila             --------------------------------------------------------------MKHDHH-QGHTHSGKGHACHHEHNS-------------
WP_012092676.1_Rhizobiales    ----------------------------------------------------------MASKTEHDHQNQSHAHSAQGHAH--DHGAV------------

                                      210       220       230       240       250       260       270       280       290       300
                              ....|....|....|....|....|....|....|....|....|....|....|....|....|....|....|....|....|....|....|
CopA_Calbicans                NEDEPNFSVNVIQQSVVVIIPNKQIINKIVDAINDMGFDCKLI--EVLPVQRSVNTKVTALIGGMTCAACANSIDSAVKELPFILESGINVVTKAAQFVL
ATP7b_Dmelanogaster           VRVILEENAGYFDYD-----PRQTDPARIASDIDDMGFECSYPGDAADPPETPASAWTNIRVVGMTCQSCVRNIEGNIGTKPGIHSIEVQLAAKNARV-Q
CopA_Hpylori                  -------------------------------------------------------------------------IERSLGRKSFVKKIEVSLLNKSANI--
CopA_like_Nvulgaris           VAVNLATEVMDVTWD------DRTDLDAIVRQVSALGFEAMPP--DAAPAGGGNDLRFA--VSGMTCAVCAGRIEKVVGTMDGVGAVSVNLAAETAQV--
CopA_Lpneumophila             ---------------------------------------------------------------------------------PKTQQASSKMEGPIVYT--
WP_012092676.1_Rhizobiales    ---------------------AQDDTHKVKDPVCGMIVDPHTAQHKAEHAGRPYYF--------CSAGCRAKFLAEPESYLDPAAAEVVPILEGTVYT--

                                      310       320       330       340       350       360       370       380       390       400
                              ....|....|....|....|....|....|....|....|....|....|....|....|....|....|....|....|....|....|....|
CopA_Calbicans                EDDGGSNIAKLKETIEDCGFDFELLSTEKVNYTSGK--------------------QKPRSINLKVNGMFCNHCPDIITQYLTNYGDAVVINDPI-----
ATP7b_Dmelanogaster           YDPAQYDPAQIAELIDDMGFEASVQEPRSPSQSPSPAPASSPKKRATPTPPPPSYAQNGSAVAIPVEQELLTKCFLHIRGMTCASCVAAIEKHCKKIYGL
CopA_Hpylori                  ----------------------------------------------------------------------------------------------------
CopA_like_Nvulgaris           -------------------------------------------------------------------------TPL------------------------
CopA_Lpneumophila             -------------------------------------------------------------------------CPMH-----------------------
WP_012092676.1_Rhizobiales    -------------------------------------------------------------------------CPMH-----------------------

                                      410       420       430       440       450       460       470       480       490       500
                              ....|....|....|....|....|....|....|....|....|....|....|....|....|....|....|....|....|....|....|....|
CopA_Calbicans                ----------------------------------------------------------------SLDHPVIKFTYVPSQTLKTRKILFDLNHLKDDGNGG
ATP7b_Dmelanogaster           DSILVALLAAKAEVKFNANVVTAENIAKSITELGFPTELIDEPDNGEAEVELEIMGMTCASCVNKIESHVLKIRGVTTASVTLLTKRGKFRYITEETGPR
CopA_Hpylori                  ------------------------------------------------------------------------------------------EFNENETNLD
CopA_like_Nvulgaris           ------------------------------------------------------------------------------------------PGVDAGSLAA
CopA_Lpneumophila             ------------------------------------------------------------------------------------------PEIRQSAPGH
WP_012092676.1_Rhizobiales    ------------------------------------------------------------------------------------------PEIRQDGPGS

                                      510       520       530       540       550       560       570       580       590       600
                              ....|....|....|....|....|....|....|....|....|....|....|....|....|....|....|....|....|....|....|....|
CopA_Calbicans                YTITPDEKGAFECEIIKPVT-------VDEHVRKLAHREVLGLAIRLAIATLFAIPTFIFGI-VAMSLLPKHHPFRIWVEDPLWVGNVSRNSWILFILAT
ATP7b_Dmelanogaster           SICEAIEALGFEAKLMTGRD-------KMAHNYLEHKEEIRKWRNAFLVSLIFGGPCMVAMIYFMLEMSDKGHAN-----MCCLVPGLSMENLVMFLLST
CopA_Hpylori                  EIFKLIEKLGYSPKKTLAEE----------------KKEFFSPNVKLALAVIFTLFV------VYLSMGAMLSPSLLPESLLTINNHSNFLNACLQLIGT
CopA_like_Nvulgaris           SVVERISGLGFGATLQEQAADAASSASLWERQRHDADARLARMKARLVPEFGFTVPL------LLLSM---GHMMGLPLPGFLHPAHSPLTFAVAQLLLT
CopA_Lpneumophila             C---PLCGMALEPETVTVSE--------------VVSPEYLDMRRRFWIALMLTIPV------VILEMG--GH-------GLKHFISGNGSSWIQLLLAT
WP_012092676.1_Rhizobiales    C---PICGMALEPVLVSLEA--------------EPNVELIDMTRRFWIGLVLAIPV------FILEMG--GHLF-----GLTHAIGQQVSNWLQLILAT

                                      610       620       630       640       650       660       670       680       690       700
                              ....|....|....|....|....|....|....|....|....|....|....|....|....|....|....|....|....|....|....|....|
CopA_Calbicans                PVYFFAADTFHRKAIKEIKSLWFHKNSFKSRLLKFGSMNLLMSLGTTVAYFASIVLIGLSATQKPMTHH--GLHTTYFDSVVFLSFFLLIGKLLQSYSKS
ATP7b_Dmelanogaster           PVQFFGGFHFYVQSYRAIKH-------------GTTNMDVLISMVTTISYVYSVAVVIAAVLLEQNSSP-----LTFFDTPPMLLIFISLGRWLEHIAKG
CopA_Hpylori                  LIVMHLGRDFYIQGFKALWH-------------RQPNMSSLIAIGTSAALISSLWQLYFVYTSQWSYGH------YYFESVCVILMFVMVGKRIENVSKD
CopA_like_Nvulgaris           LPVMWSGRDFYRIGFGNLRR-------------LSPNMDSLVALGTGAAFIYSLWNTVEIALGVDVAHR---VMDLYYESAAVLITLVSLGKYFEMRSRA
CopA_Lpneumophila             PVVLWGGWPFFKRGWQSLKT-------------GQLNMFTLIAMGIGVAWIYSMVAVLWPGVFPHAFRSQEGVVAVYFEAAAVITTLVLLGQVLELKARE
WP_012092676.1_Rhizobiales    PVVLWAGWPFFQRGWQSLVN-------------RSLNMFTLIAMGTGTAWIYSVVATLAPGIFPDAFRQHDGSVAVYFEAAAVITVLVLLGQVLELRARE

                                      710       720       730       740       750       760       770       780       790       800
                              ....|....|....|....|....|....|....|....|....|....|....|....|....|....|....|....|....|....|....|....|
CopA_Calbicans                KTADAISELSKLKQTEATLI----------EDNKD------TRVDIQLLEIGDQIRIGTGESPPVDCVLVEGSTEFDESALTGESTPVKHQEGHQIFSGT
ATP7b_Dmelanogaster           KTSEALSKLLSLKAADALLV----------EISPDFDIISEKVISVDYVQRGDILKVIPGAKVPVDGKVLYGHSSCDESLITGESMPVAKRKGSVVIGGS
CopA_Hpylori                  KALDAMQALMKNAPKTALKM----------HNNQQ------IEVLVDSIVVGDILKVLPGSAIAVDGEIIEGEGELDESMLSGEALPVYKKVGDKVFSGT
CopA_like_Nvulgaris           RTSEAIKSLMDLAPETALRLVSADGGPAVGHEAGDGTNVAVQEVPVAEVRAGDLLQVRPGARIPVDGTVVSGTSSVDESMLTGESLPVSKTGGDSVAGGT
CopA_Lpneumophila             QTGSAIRALLKLVPESAHRI----------KEDGS-----EEEVSLDNVAVGDLLRVRPGEKIPVDGEVQEGRSFVDESMVTGEPIPVAKEASAKVIGAT
WP_012092676.1_Rhizobiales    STSGAIRALLDLAPKTARII----------RDDGT-----EEEVQLDTVRVGDRLRVRPGEKVPVDGEVLEGRSAVDESMVTGESMPVTKEVGATAIGGT

                                      810       820       830       840       850       860       870       880       890       900
                              ....|....|....|....|....|....|....|....|....|....|....|....|....|....|....|....|....|....|....|....|
CopA_Calbicans                VNIGNSSVVGKVTSLEGDSLLNQIVNTVRDGQMRKAPMERTADLITGYFVPLIIACAVITWVIWLALGYSGA------------LPQSYLDIDVGGWTVW
ATP7b_Dmelanogaster           IN-QNGVLLVEATHTGENTTLAQIVRLVEEAQTSKAPIQQLADRIAGYFVPFVVVVSSITLIAWIIIGFSNPNLVPVAMEHKMHMDQNTIIVSYA-----
CopA_Hpylori                  FN-SHTSFLMKATQDNKNSTLSQIVEMIHNAQSSKAEISRLADKVSSVFVPSVIAIAILAFVVWLIIA-----------------PKPDFWWNFG----I
CopA_like_Nvulgaris           IN-RLGTFVMRAERVGADTVLARIIRLVEEAQGSKAPIANIADRVSLYFVPTVMALAVLAGVGWYTVG------------------DADF--TFA-----
CopA_Lpneumophila             IN-QTGSFVMKALHVGSDTMLARIVQMVSDAQRSRAPIQRLADTVSGWFVPAVILVAVLSFIVWALLG-----------------PQPAL--SYG-----
WP_012092676.1_Rhizobiales    MN-QSGALVIEAKKVGRDTMLSQIVQLVAEAQRSRAPIQRLADQVSGWFVPAVIVVAILAFITWSIWG-----------------PEPRF--SFG-----

                                      910       920       930       940       950       960       970       980       990      1000
                              ....|....|....|....|....|....|....|....|....|....|....|....|....|....|....|....|....|....|....|....|
                                [TMD 6 CxC motif]
CopA_Calbicans                SLEFAIAVFVVACPCGIGLAAPTALFVGSGLAAKNGILAKGGGVAFQDGANVNIVCFDKTGTLTKGELTITDFHFPD-----------------------
ATP7b_Dmelanogaster           -FKCALSVLAIACPCALGLATPTAVMVATGTGAINGVLVKGAT-ALENAHKVKTVVFDKTGTITHGTPMTSKVTLFVTAQ--------------------
CopA_Hpylori                  ALEVFVSVLVISCPCALGLATPMSILVANQKASSLGLFFKDAK-SLEKARLVNTIVFDKTGTLTNGKPVVKSVHS-------------------------
CopA_like_Nvulgaris           -LRIFVAVMVIACPCAMGLATPTSIMVGTGRGAQLGILVKSGA-ALETAGRVDTVVFDKTGTLTEGKPRLVHVSAVEDGPWQTDVSASGGDGTQGGASGM
CopA_Lpneumophila             -LIAAVSVLIIACPCALGLATPMSIMVGVGKGAQSGVLIKNAE-ALERMEKVNTLVVDKTGTLTEGHPKLTRIV--------------------------
WP_012092676.1_Rhizobiales    -LIAAVSVLIIACPCALGLATPMSIMVGVGRGAQAGVLIKNAE-ALEHMEKVDTIIVDKTGTLTEGRPAVTAIVP-------------------------

                                     1010      1020      1030      1040      1050      1060      1070      1080      1090      1100
                              ....|....|....|....|....|....|....|....|....|....|....|....|....|....|....|....|....|....|....|....|
CopA_Calbicans                ------KSSIAKTF----ALQVARDLEVPSHHPLGKAVKSFVTSYDGKSLTA-----NKIPQVETVPGKGLR----------------------------
ATP7b_Dmelanogaster           ------VCSLAR------ALTIVGAAEQNSEHPIASAIVHFAKDMLNVGATPQAGSFGKSSHFQAVPGCGIRVTVSNYEQTLRQACNADRIINYENLHRT
CopA_Hpylori                  ------NIELLE------LLSLAGSIEKSSEHVIAKGIVEYAKE---HNAPL-----KEMSEVKVKTGFGIS----------------------------
CopA_like_Nvulgaris           RLTQPLAPDVAEDTPRRMVLRLAASLEAVSEHPLAEAILAGAAE---AGIAP-----WPVEAFEAVPGRGVR----------------------------
CopA_Lpneumophila             ------TDDFVEDN----ALALAAALEHQSEHPLANAIVHAAKE---KGLSL-----GSVEAFEAPTGKGVV----------------------------
WP_012092676.1_Rhizobiales    ------APGFTEDE----ALRLAASVERASEHPLALAIVRAAEE---RGIVT-----APVADFDSPTGKGAY----------------------------
```

```
                                  1110      1120      1130      1140      1150      1160      1170      1180      1190      1200
                            ....|....|....|....|....|....|....|....|....|....|....|....|....|....|....|....|....|....|....|....|
CopA_Calbicans              ---GQIIYDEGDDIWN-------------------------------------EYKPTQAILGNEKLMEENDIE--FTEKERQCLKKWKGECKSVILVGL
ATP7b_Dmelanogaster         HPQGSVPVDNGASIEHLLPQRSVRKSMELNNQQLLSDLVLEPEEELLTDQKIIDSPEILVLIGNREWMERNAIE--VPLEISDCMTHEERKGHTAVLCAL
CopA_Hpylori                ---AKTDYQGAKEV---------------------------------------------IKVGNSEFFNPINALEIQENGILVFVGR--------VISEK
CopA_like_Nvulgaris         ---GRVRTDAGESG---------------------------------------------VLLGNHAFMAEAGVAGLDAHGLREMLDALADAGVTPLLLAA
CopA_Lpneumophila           ---GQVDGHH-------------------------------------------------VAIGNARLMQEHGG---DNAPLFEKADELRGKGASVMFMAV
WP_012092676.1_Rhizobiales  ---GTVEGKR-------------------------------------------------IPLGNAKFLAEQGV---DVAPLADEADRLREDGATAIFMGV

                                  1210      1220      1230      1240      1250      1260      1270      1280      1290      1300
                            ....|....|....|....|....|....|....|....|....|....|....|....|....|....|....|....|....|....|....|....|
CopA_Calbicans              QCESYFKDSKFHLALIMAARDQLRPETKKVIEFLKHKDIECWMITGDNKLTADAIAKEIGIENVVSEVLPDEKEAQIKKIRQLNSGGKKEPVIAMIGDGI
ATP7b_Dmelanogaster         NGQ---------LVCMFAVSDMVKPEAHLAVYTLKRMGIDVVLLTGDNKNTAASIAREVGIRTVYAEVLPSHKVAKIQRIQA------NGIRVAMVGDGV
CopA_Hpylori                EDE---------LLGAFVLEDLPKKGVKEHIAQIKKLGINTFLLSGDNRENVKKCALELGIDGYISNAKPQDKLNKIKELKE------KGQIVMMVGDGL
CopA_like_Nvulgaris         AGE---------MRGIVGVADPLRAEARGVLERLRQCGVRAVMLTGDNRRTAEAIARQAGMDEVVAEVMPDAKEREVSRLQG------EGRVVAMVGDGI
CopA_Lpneumophila           DGK---------TVALLVVEDPIKSSTPETILELQQSGIEIVMLTGDSKRTAEAVAGTLGIKKVVAEIMPEDKSRIVSELKD------KGLIVAMAGDGV
WP_012092676.1_Rhizobiales  DNR---------IAAIFAIADPVKPSTPEALAALKAQGIRVVMLTGDNWTTAKAVARRLGIDEVEAEVLPDQKSAVVQRHKA------AGEVVAMAGDGV

                                  1310      1320      1330      1340      1350      1360      1370      1380      1390      1400
                            ....|....|....|....|....|....|....|....|....|....|....|....|....|....|....|....|....|....|....|....|
                                                                                        TMD 7 YN(X4)P motif       TMD 8 MXXSS motif
CopA_Calbicans              NDAPALASADVGIALSSGADLAVTSSDFILLNKMHPLVSLATLLDICSKVFQRVKLNFGWSLVYNLIALPIAAGVIYPYKNSRLDPVWASAAMALSSVSV
ATP7b_Dmelanogaster         NDSPALAQADVGITIAAGTDVAAEASDIVLMR--NDLLDVVACLDLSRCTVRRIRYNFFFASMYNLLGIPLASGLFAPY-GFTLLPWMASVAMAASSVSV
CopA_Hpylori                NDAPSLAMSDVAVVMAKGSDVSVQAADIVSFN--NDIKSVYSAIKLSQATIKNIKENLFWAFCYNSVFIPLACGVLYKA-NIMLSPAIAGLAMSLSSVSV
CopA_like_Nvulgaris         NDAPALARADVGIAMGTGIDVAVEAGDIVLLR--GGLTSVPVAMQLSRATMRNIRQNLFWAFGYNVLGIPVAAGLLYAFGGPTLSPMLAGAAMALSSVSV
CopA_Lpneumophila           NDAPALAKADIGIAMGTGTDVAIESAGVTLLH--GDLRGIAKARRLSESTMSNIRQNLFFAFIYNVLGVPLAAGVLYPLTGLLLSPMIAAAAMALSSVSV
WP_012092676.1_Rhizobiales  NDAPALAAADVGIAMGTGTDVAMESAGVTLLK--GDLTGIVRARRLSEAVMRNIRQNLFFAFIYNALGVPVAAGVLYPFFDILLSPIIAAAAMALSSVSV

                                  1410      1420      1430      1440      1450      1460      1470      1480      1490      1500
                            ....|....|....|....|....|....|....|....|....|....|....|....|....|....|....|....|....|....|....|....|
CopA_Calbicans              VLSSLALKLYK--------------------------------PSLKAEDFGVDDSTESELSPNEV----------------------------------
ATP7b_Dmelanogaster         VCSSLLLKMYRKPTAKTLRTAEYEAQLAAERASGSEDELDKLSLHRGLDDLPEKGRMPFKRSSTSLISRIFMHDYGNITSPDAKYGEGLLDPEEQYDGRT
CopA_Hpylori                VLNSQRLRNFK--------------------------------IKDH-----------------------------------------------------
CopA_like_Nvulgaris         VGNALRLRLFT--------------------------------PERGGTC--------------------------------------------------
CopA_Lpneumophila           IINALRLKRVT--------------------------------L--------------------------------------------------------
WP_012092676.1_Rhizobiales  IANASRLRVTR--------------------------------L--------------------------------------------------------

                                  1510
                            ....|....|....|...
CopA_Calbicans              ------------------
ATP7b_Dmelanogaster         KIVRSRFHANDSTELQKL
CopA_Hpylori                ------------------
CopA_like_Nvulgaris         ------------------
CopA_Lpneumophila           ------------------
WP_012092676.1_Rhizobiales  ------------------
```

Figure S1.Multiple amino acid sequence alignment of Cu(I) ATPases. The three key amino acid signature motifs (CXC, YN(X)4P, and MSXST) are red-highlighted.

A)

```
Brucella anthropi ATCC 49188    1901 TGCTTACGGGTGATAACTGGACCACGGCCAAGGCGGTCGCCCGCCGGCTC   1950
                                     ||||||||||||||||||||||||||||||||||||||||.|||||||||
Aureimonas sp. OT7              1901 TGCTTACGGGTGATAACTGGACCACGGCCAAGGCGGTCGCGCGCCGGCTC   1950
                                        L  T  G  D  N  W  T  T  A  K  A  V  A  R  R  L
```

B)

```
Brucella anthropi ATCC 49188    1901 TGCTTACGGGTGATAACTGGACCACGGCCAAGGCGGTCGCCCGCCGGCTC   1950
                                     ||||||||||||||||||||||||||||||||||||||||.|||||||||
Aureimonas altamirensis A31     1901 TGCTTACGGGTGATAACTGGACCACGGCCAAGGCGGTCGCGCGCCGGCTC   1950
                                        L  T  G  D  N  W  T  T  A  K  A  V  A  R  R  L
```

Figure S2. Identification of point mutations in the genes encoding the Ms-Cu(I)-ATPase.

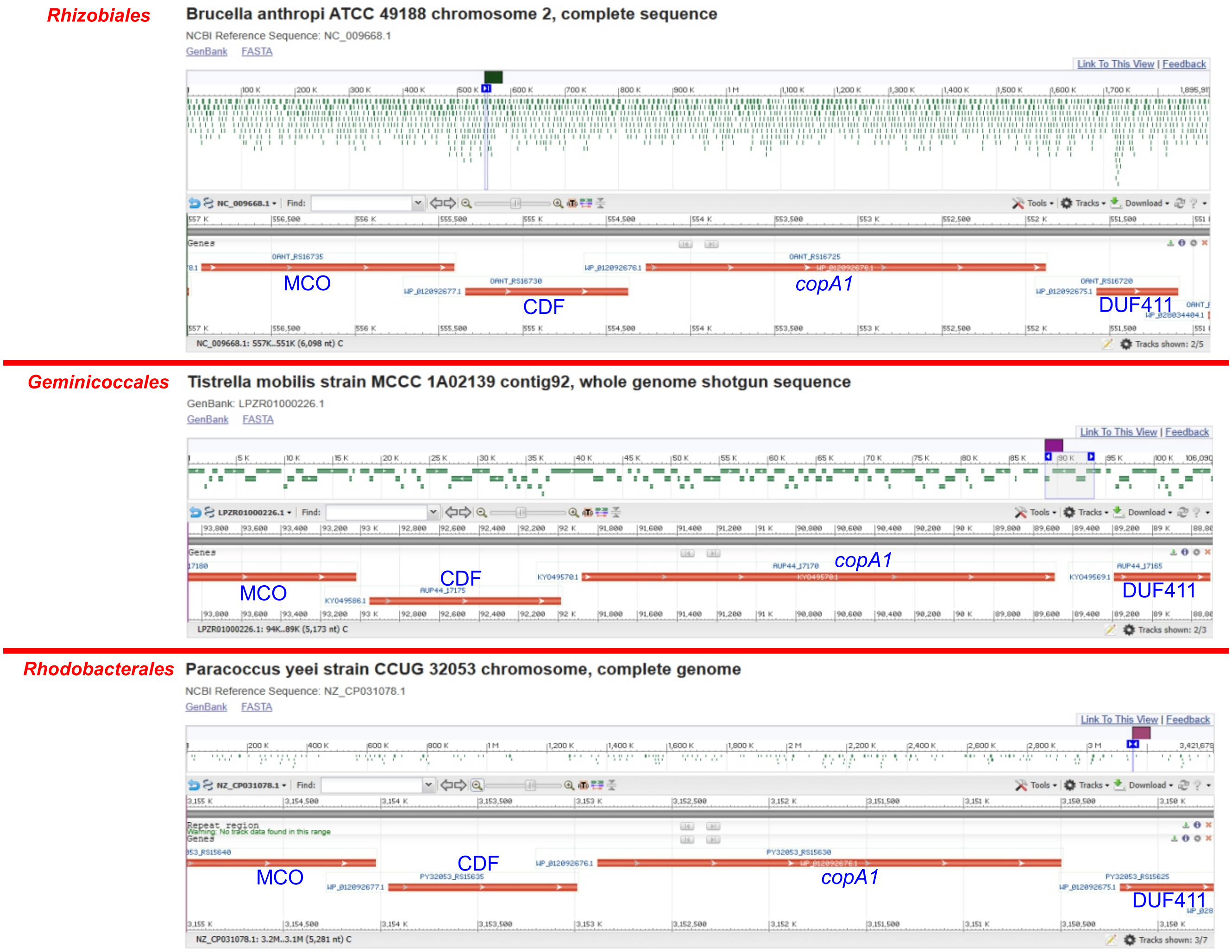

Supplementary Figure S3. Screenshot of the gene context of the Ms-Cu(I) ATPase (WP_012092676.1) displayed by the Graphics tool (NCBI). It shows a collinear organization with the other three genes involved in metal homeostasis and is conserved across multiple orders within the class Alphaproteobacteria, including *Brucella anthropi* ATCC 49188 (Rhizobiales order), *Tistrella mobilis* MCCC 1A02139 (Geminicoccales order), and *Paracoccus yeei* CCUG 32053 (Rodobacterales order).

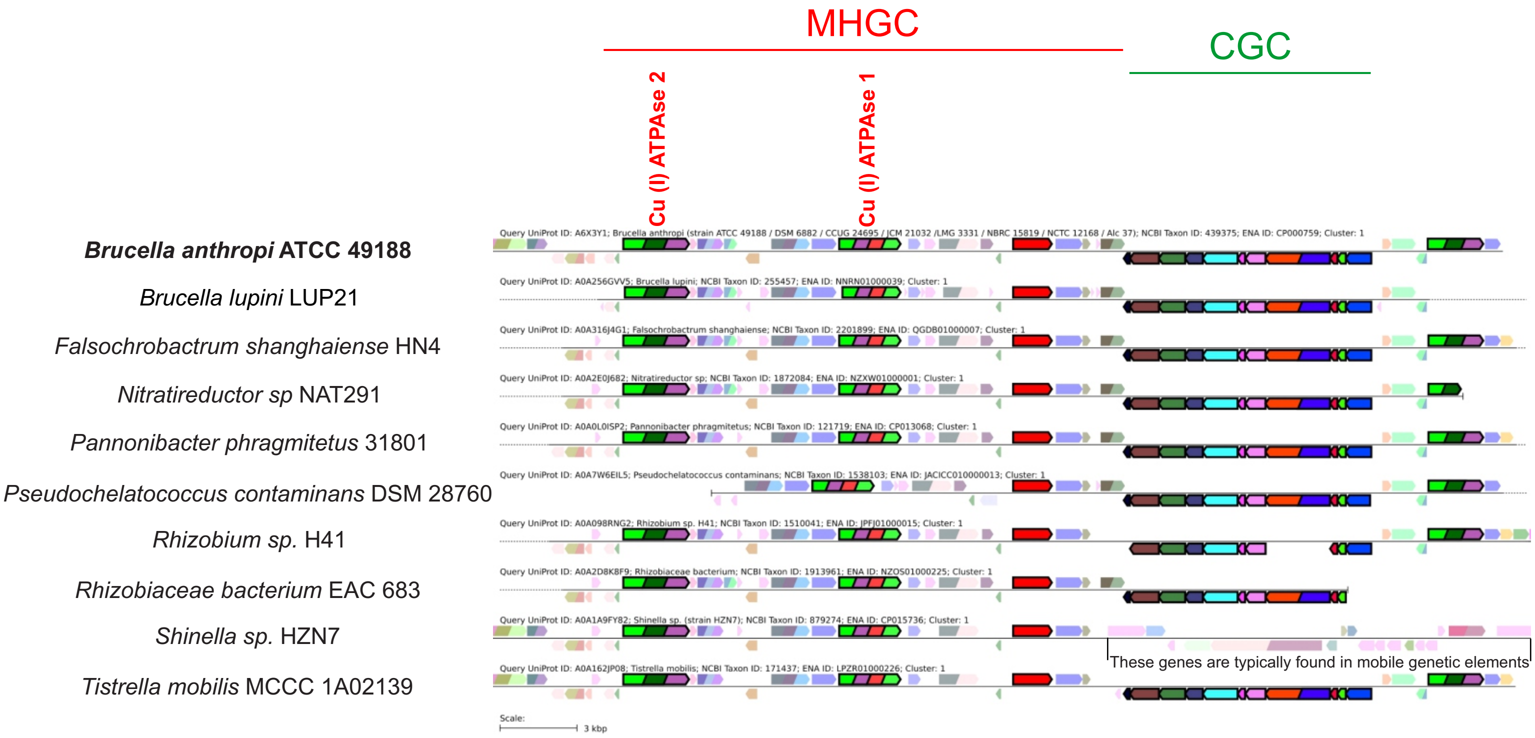

Figure S4. Loss of collinear organization.The conjugation gene cluster (CGC) in *Shinella sp*. HZN7 was replaced by a 25-kb gene cluster encoding transposases, relaxases, methylases, a toxin-antitoxin stability system, endonucleases, and integrases, resembling components of mobile genetic elements (MGEs).

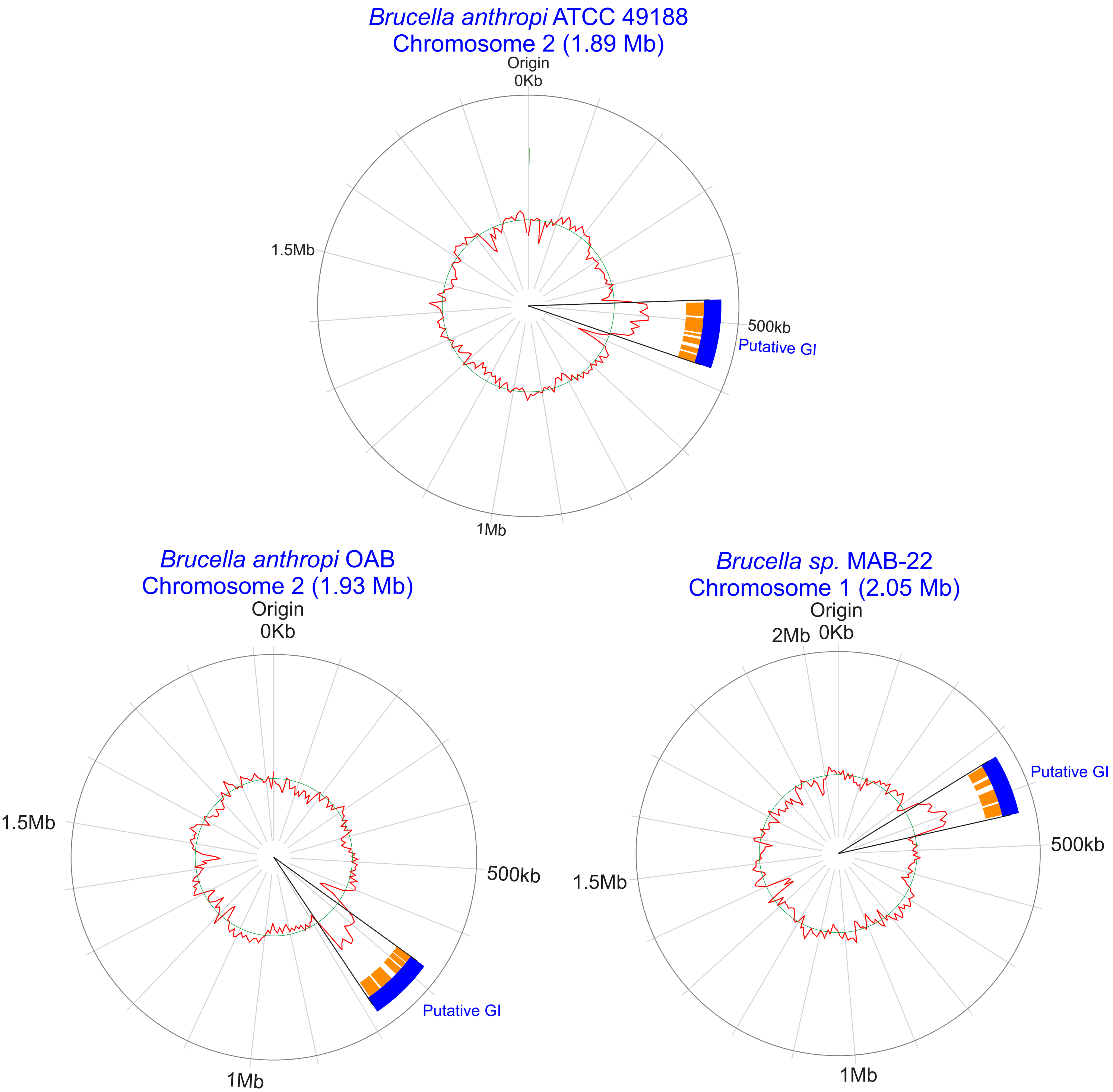

Figure S5. Multiple *Brucella* strains have putative ~110 kb GIs in their genomes. (A) *Brucella anthropi* ATCC 49188 GI is located on chromosome 2. (B) *Brucella anthropi* OAB GI is also located on chromosome 2, and (C) *Brucella sp*. MAB-22 GI is located on chromosome 1.

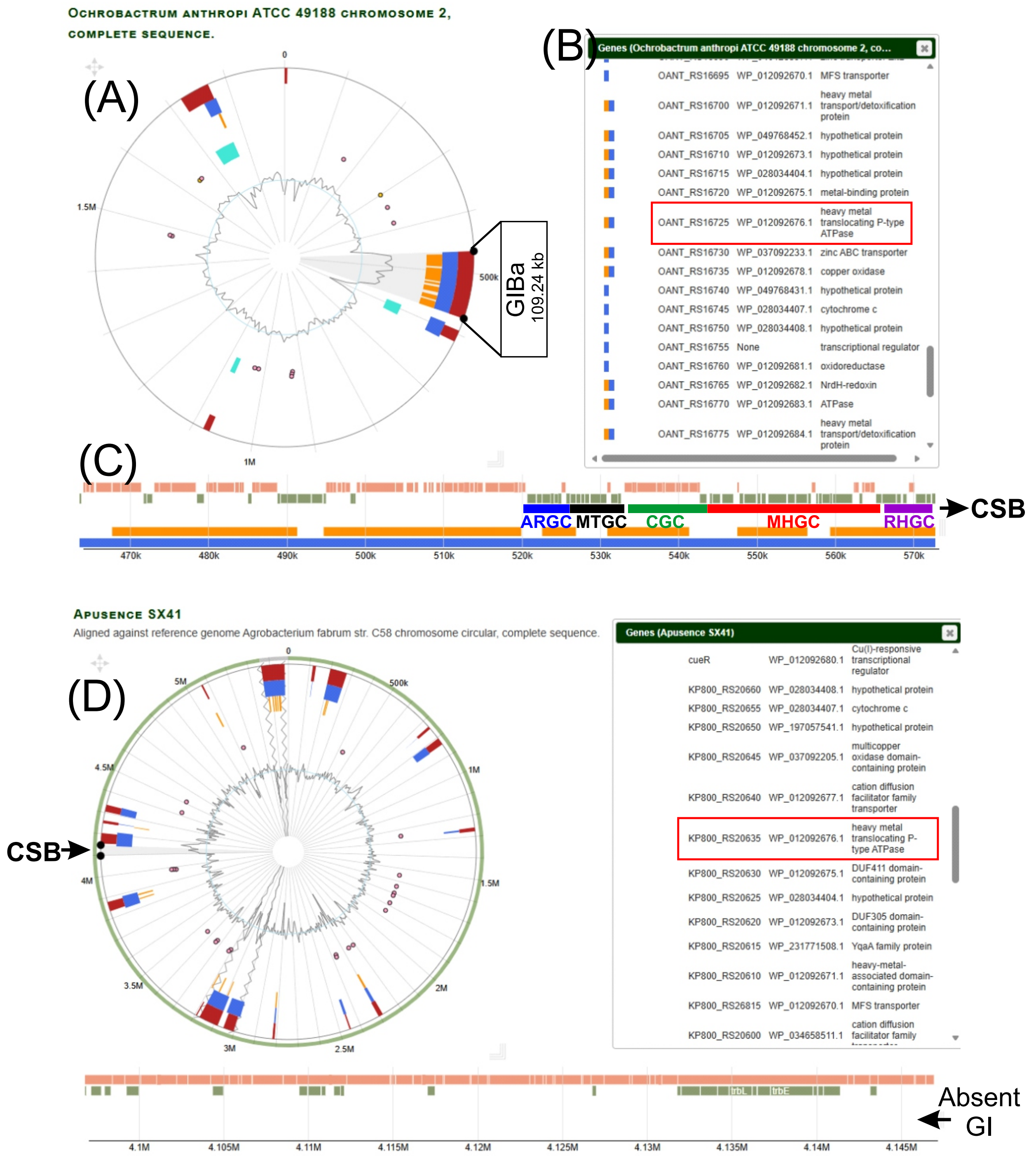

Figure S6. (A) A screenshot of the IslandViewer4 output shows the nucleotide composition bias that aligns with the predicted GI >100 kb on chromosome 2, as detected by the IslandPath -DIMOB predictor (continuous blue line) and the SIGI_HMM predictor (discontinuous orange lines). (B) Shows a s2s2 of genes contained in the GI and their encoded proteins. (C) Genetic map of the 109.246 kb GI and the location of the CSB. (D) The CSBs encoded in the genomes of *Agrobacterium pusence* SX41 and three strains of *Aureimonas altamirensis* are not located within a GI.

Figure S7. ***Brucella anthropi*** **ATCC 49188 chromosome 2, complete sequence.**

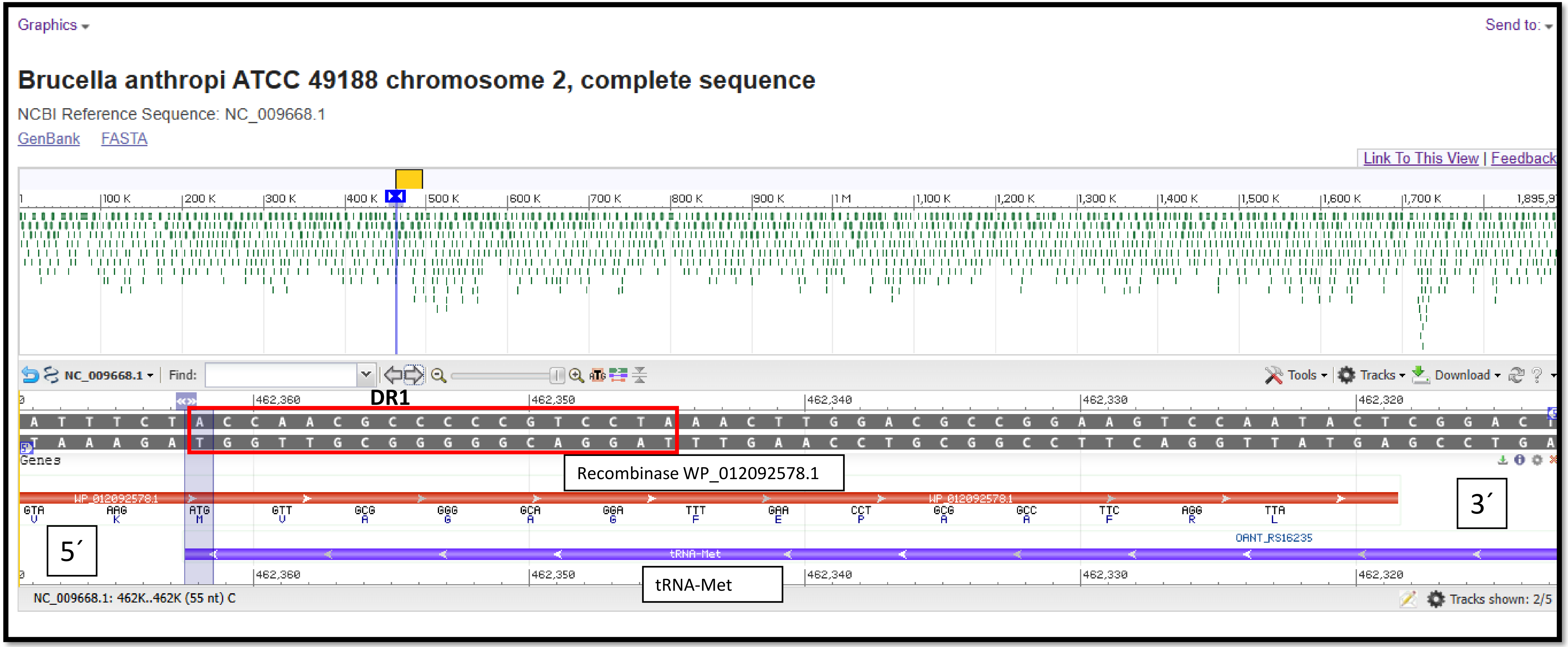

Supplement: Supplementary file 1 [file microorganisms-14-02390-s001.zip › microorganisms-4483781-Supplementary Figures.pdf]
